# Ten‐Year Cost‐Effectiveness of the Risk Assessment and Management Programme—Diabetes Mellitus (RAMP‐DM) Relative to Usual Care in Hong Kong Public Primary Care: A Population‐Based Cohort Study

**DOI:** 10.1111/dom.71242

**Published:** 2026-08-23

**Authors:** Eric Ho Man Tang, Ivy Lynn Mak, Emily Tsui Yee Tse, Esther Yee Tak Yu, Julie Yun Chen, Weng Yee Chin, David Vai Kiong Chao, Wendy Wing Sze Tsui, Tony King Hang Ha, Carlos King Ho Wong, Cindy Lo Kuen Lam, Eric Yuk Fai Wan

**Affiliations:** ^1^ Department of Family Medicine and Primary Care, Li Ka Shing Faculty of Medicine The University of Hong Kong Hong Kong SAR China; ^2^ School of Public Health, Li Ka Shing Faculty of Medicine The University of Hong Kong Hong Kong SAR China; ^3^ Primary Healthcare Commission Health Bureau, The Government of the Hong Kong Special Administrative Region Hong Kong SAR China; ^4^ Department of Family Medicine and Primary Health Care United Christian Hospital and Tseung Kwan O Hospital, Hospital Authority Hong Kong SAR China; ^5^ Department of Family Medicine and Primary Healthcare Queen Mary Hospital, Hong Kong West Cluster, Hospital Authority Hong Kong SAR China; ^6^ Strategy and Planning Division Head Office, Hospital Authority Hong Kong SAR China; ^7^ Leicester Real World Evidence Unit, Diabetes Research Centre, College of Life Sciences University of Leicester Leicester UK; ^8^ Centre for Safe Medication Practice and Research, Department of Pharmacology and Pharmacy, Li Ka Shing Faculty of Medicine The University of Hong Kong Hong Kong SAR China; ^9^ Comprehensive Primary Healthcare Collaboratory, Li Ka Shing Faculty of Medicine The University of Hong Kong Hong Kong SAR China; ^10^ The Institute of Cardiovascular Science and Medicine, Li Ka Shing Faculty of Medicine The University of Hong Kong Hong Kong SAR China

**Keywords:** cost‐effectiveness, direct medical cost, multidisciplinary programme, primary health care, type 2 diabetes mellitus

## Abstract

**Aims:**

The Risk Assessment and Management Programme—Diabetes Mellitus (RAMP‐DM) is a multidisciplinary, protocol‐driven, risk‐stratified care model integrated into public primary care in Hong Kong for patients with type 2 diabetes mellitus (T2DM). This study evaluated the real‐world 10‐year cost‐effectiveness of RAMP‐DM from the public healthcare provider perspective.

**Materials and Methods:**

Programme costs from 2009 to 2019 were estimated using costing questionnaires and reported in 2019 US dollars. Direct medical costs were compared between patients enrolled in RAMP‐DM and a propensity‐score matched cohort receiving usual care only. Effectiveness was based on previously published 10‐year estimates of absolute risk reduction in complications and all‐cause mortality. The primary outcome was the incremental total direct medical cost per diabetes‐related complication avoided over 10 years. Secondary outcomes included incremental cost per all‐cause mortality avoided and programme cost per complication avoided. Sensitivity analyses examined the effects of parameter uncertainty and discounting.

**Results:**

A total of 36 746 patients (18 373 in each group) were included. RAMP‐DM was associated with absolute risk reductions of 12.1% for any complications and 16.2% for all‐cause mortality. Accounting for the programme cost of US$329 per participant, total undiscounted direct medical costs over 10 years was US$10 543 lower per person among RAMP‐DM participants (RAMP‐DM: $23 430; usual care: US$33 973). RAMP‐DM was a cost‐saving strategy relative to usual care.

**Conclusions:**

RAMP‐DM was associated with better 10‐year clinical outcomes and lower direct medical costs. These findings support the economic value of structured multidisciplinary diabetes management programmes in publicly funded primary care settings, although residual confounding and uncertainty in cost estimates should be considered.

## 1 Introduction

Type 2 diabetes (T2DM) remains a public health challenge. In Hong Kong, approximately 8.5% of the population has diabetes and annual diabetes‐related healthcare expenditure was approximately US$254 million. Globally, T2DM‐related expenditure is projected to exceed US$1054 billion by 2045 [1].

Integrated chronic disease management models based on the Chronic Care Model have consistently improved the quality of diabetes care and clinical outcomes through coordinated chronic disease management, team‐based care, evidence‐based guidelines and support for patient self‐management [2, 3, 4, 5, 6, 7, 8].

The Risk Assessment and Management Programme—Diabetes Mellitus (RAMP‐DM) is a territory‐wide diabetes management programme introduced in 2009 by the Hong Kong Hospital Authority (HA), the main public healthcare service provider in Hong Kong. RAMP‐DM supplements usual primary care in public General Outpatient Clinics (GOPCs) with structured risk assessment, protocol‐driven risk stratification, multidisciplinary management involving primary care physicians, nurses and allied health professionals (optometrists, dietitians, podiatrists and physiotherapists) and patient education [9, 10]. Patients receive regular follow‐up under the RAMP‐DM in addition to usual care every 2–4 months in GOPCs with reassessments guided by initial risk status and glycemic control. Previous evaluations have demonstrated that participation in RAMP‐DM was associated with improved outcomes and lower direct medical costs after 5 years [11, 12, 13, 14, 15, 16, 17, 18], although the reduction in complications appeared to attenuate after 8–9 years of follow‐up [18].

Despite growing evidence supporting multidisciplinary diabetes management, long‐term economic evidence from real‐world practice remains limited and the economic value of chronic disease management programmes may change over time due to ongoing requirement for staff training, equipment replacement and programme expansion.

Several landmark randomised controlled trials have evaluated the cost‐effectiveness of integrated diabetes care with mixed results. The Steno‐2 study found that intensive multifactorial intervention with lifestyle modification and pharmacological treatment for patients with T2DM and microalbuminuria was cost‐effective over 10 years (€2538 per QALY gained) [19], with associated with 14.5% lower long‐term healthcare than conventional treatment [20]. Similarly, the SPLINT trial reported that integrated specialist nurse‐led with antihypertensive and lipid‐lowering treatment was cost‐effective, with a lifetime incremental cost‐effectiveness ratio of US$9070 per QALY gained [21]. However, the ADDITION trials found that multidisciplinary interventions for screen‐detected T2DM were not cost‐effective over 10 years [22, 23]. Existing economic evaluations often relied on simulation modelling, trial populations, or intermediate outcomes rather than observed long‐term outcomes in routine practice. This study addresses these gaps by providing a real‐world 10‐year economic evaluation of RAMP‐DM using empirical programme costs, healthcare utilisation and observed clinical outcomes within Hong Kong's public primary healthcare system.

## 2 Methods

### 2.1 Study Design, Setting and Participants

#### 2.1.1 Study Design

This economic evaluation was planned a priori within the broader RAMP‐DM evaluation programme, combining empirical costing data collected retrospectively with 10‐year effectiveness and healthcare utilisation data from a previously established propensity‐score matched cohort [16].

A cost‐effectiveness analysis was conducted from the public healthcare providers' perspective between 2009 and 2019. This perspective was chosen because RAMP‐DM is funded and delivered within the Hong Kong public healthcare system and the findings were intended to inform healthcare resource allocation and policy decisions.

Cost‐effectiveness was evaluated by comparing incremental total direct medical costs against incremental effectiveness. The primary effectiveness outcome was the between‐group difference in the cumulative incidence of any diabetes‐related complication over 10 years. The primary economic outcome was the incremental total direct public medical cost per diabetes‐related complication avoided. Secondary outcomes included cost per all‐cause mortality avoided and programme cost per complication avoided as an implementation‐focused metric. When total costs were lower and outcomes better, RAMP‐DM was considered cost‐saving/economically dominant relative to usual care.

#### 2.1.2 RAMP‐DM Setting

Detailed descriptions of RAMP‐DM have been reported previously [9, 15, 24]. Briefly, all enrolled patients undergo a nurse‐led comprehensive assessment covering cardiovascular risk factors, glycemic control, complications and lifestyle behaviours. Patients are then stratified into four risk categories according to predefined criteria adapted from the Joint Asia Diabetes Evaluation programme [9, 25].

Subsequent management is tailored according to risk level and delivered by a multidisciplinary team comprising primary care physicians, nurses and allied health professionals. All participants are referred to the Patient Empowerment Program to support self‐management and lifestyle modifications. Patients with suboptimal glycemic control (HbA1c > 7.0%) receive structured nurse follow‐up. Referrals to dietitians, podiatrists, physiotherapists, ophthalmology services, wound clinics, smoking cessation or patient support call services are made according to clinical need. Patients at particularly high risk may be referred for more intensive specialist‐led management. Follow‐up frequency is guided by baseline risk and service availability. All RAMP‐DM participants continue to receive routine diabetes care in GOPCs. Reassessment is typically performed every 2–3 years for clinically stable patients.

#### 2.1.3 Usual Care

Patients receiving usual care continue to receive physician‐led diabetes care in GOPCs. Consultations typically occur every 2–3 months, last 6–7 min and include routine clinical review, medication management, laboratory monitoring and lifestyle advice. Availability of medication, access to laboratory tests, investigations and allied health services were similar between groups. Usual care does not include structured nurse‐led risk assessment, risk stratification, predefined multidisciplinary referral pathways, or programme‐specific support services.

#### 2.1.4 Study Population

Patients with T2DM who completed a RAMP‐DM assessment between 1 August 2009 and 30 September 2010 were included in the analysis. Adult T2DM patients who had never attended any RAMP‐DM services and received usual care only during the study period were included as comparison group.

All participants had no known diabetes‐related complications at baseline, including cardiovascular diseases (CVD, composite of coronary heart disease, heart failure, stroke and peripheral vascular disease), end‐stage renal disease (ESRD), sight‐threatening diabetic retinopathy, maculopathy, peripheral neuropathy or estimated glomerular filtration rate < 45 mL/min/1.73 m^2^ at baseline. Incidences of diabetes‐related complications, all‐cause mortality and public healthcare utilisation from 2009 to 2019 were compared between RAMP‐DM participants and propensity score‐matched patients receiving usual care only.

### 2.2 Costing Analysis of RAMP‐DM

The HA manages 74 GOPCs within seven geographical clusters. Costs incurred in implementing and operating RAMP‐DM were collected using structured costing questionnaires at three administrative levels: (1) HA Head Office, (2) geographical cluster and (3) two randomly selected clinics within each cluster. At the Head Office level, questionnaires were completed by the accounting manager; at the cluster level, by the cluster Chief of Service for Primary Care; and at the clinic level, by nurse manager responsible for RAMP‐DM. Completed questionnaires were reviewed for completeness and validity. Missing or unclear responses were clarified with respondents and all data were double‐entered before analysis.

Costs were categorised into fixed set‐up costs and recurrent costs. Fixed set‐up costs comprised one‐off expenses required to set up the programme, including IT system development, staff training, equipment, furniture and renovation costs. Recurrent cost represents ongoing operational expenses, including staff costs and resources used in programme delivery. Only resources specifically attributable to RAMP‐DM were included. For personnel involved in both RAMP‐DM and other clinic activities, only the proportion of time dedicated to RAMP‐DM‐related work was allocated to programme costs. Because RAMP‐DM was implemented within existing healthcare infrastructure, institutional overhead costs, including building space, utilities and general support services, were not separately allocated.

Set‐up costs were evaluated over the 10‐year period from 2009 to 2019 and divided into two 5‐year phases: the initial implementation phase (2009 to 2014) and an expansion phase (2014 to 2019). The initial phase reflected resources required to establish the programme, whereas the expansion phase reflected additional investments required for programme scale‐up, including staff training/re‐training and replacement or procurement of equipment.

For each phase, the fixed set‐up cost per participant was calculated by dividing total set‐up cost by the number of patients enrolled during that period. Formal annualisation based on assumed useful life was not performed. The overall average fixed set‐up cost per participant was calculated as the mean of the two phase‐specific costs, assuming equal weighting of the implementation stages. A sensitivity analysis weighting costs according to the number of participants enrolled during each phase was also performed.

Recurrent costs were estimated using data from the 2018–2019 financial year. Costs were calculated separately for initial assessments, nurse‐led interventions and doctor interventions and administrative costs at both the Head Office and clinic levels. Unit costs were calculated on a per‐attendance basis, while annual administrative costs were divided by the number of RAMP‐DM participants served to derive a cost per patient.

Staff costs were estimated using the 2018/19 HA General Pay Scale. The hourly salary rate was calculated assuming that a staff member, on average, works 39 h per week and for a total of 52 weeks annually.

Annual programme cost of RAMP‐DM comprised fixed set‐up cost, total intervention cost derived by multiplying attendance frequencies by corresponding unit costs and programme administrative costs.

### 2.3 Annual Public Direct Medical Cost Associated With Health Service Utilisation

Public direct medical costs were estimated using the propensity‐score matched cohort previously employed in the 10‐year effectiveness evaluation of RAMP‐DM [18]. Public healthcare utilisation data were extracted from the HA electronic health record system. Direct medical costs were calculated by multiplying attendance frequency by corresponding unit costs, obtained from the Government Gazette (2017–2023). Unit costs covered included investigations and medications provided during each encounter [26]. A detailed breakdown of the unit costs is presented in Table S1.

For participants enrolled in RAMP‐DM, programme‐specific costs were included as an additional component of direct medical expenditure. These comprised fixed set‐up costs, intervention costs and administrative costs attributable to programme delivery.

### 2.4 Statistical Analyses

Clinical effectiveness estimates were obtained from a previously propensity‐score matched cohort evaluating the 10‐year effectiveness of RAMP‐DM [18] and applied directly as effectiveness inputs for the present economic evaluation.

Missing baseline data were handled using multiple imputation by chained equations [27, 28]. Five imputed datasets were generated using all available baseline variables and combined using Rubin's rule [29]. Patients in the RAMP‐DM and usual care groups were then matched 1:1 using propensity scores estimated from logistic regression incorporating sex, age, smoking status, public assistance status, clinical (diabetes duration, hypertension), laboratory, the Charlson Comorbidity Index and medication use. Matching without replacement was performed using the Stata package ‘psmatch2’ [30] with a calliper width of 0.001. Balance between groups was assessed using absolute standardised mean difference (ASMD), with values > 0.1 implying imbalance between groups [31].

Differences in clinical outcomes were expressed as absolute risk reduction (ARR) calculated from cumulative incidence estimates and the number needed to treat (NNT) calculated as the reciprocal of the ARR.

Healthcare utilisation and direct medical costs were summarised as means with standard deviation (SD) or range as appropriate. Differences between groups were assessed using generalised linear models with a Poisson family, log link and robust standard errors. Incidence rate ratios (IRR) for healthcare utilisation were estimated using negative binomial regression models with person‐time included as an offset.

The primary economic outcome was the incremental total direct public medical cost per diabetes‐related complication avoided over 10 years. Secondary outcomes included incremental total direct public medical cost per all‐cause mortality avoided. Programme cost per complication or death avoided was estimated as implementation cost metrics. RAMP‐DM was considered economically dominant when associated with both lower costs and better outcomes than usual care.

Costs were collected in Hong Kong Dollars (HKD) and reported as 2019 USD using an exchange rate of US$1 = HK$7.8. The primary analysis reported undiscounted costs and outcomes to reflect real‐world expenditures and clinical consequences over the follow‐up period. Sensitivity analyses were conducted including: (1) applying annual discount rates of 3% and 5% to both costs and outcomes; (2) varying the recurrent cost by ±10% and ±20%; and (3) one‐way sensitivity analysis to estimate the effect of parameter uncertainties on total direct medical costs. Analyses were performed using Stata 14.0 (StataCorp LP, College Station, Texas). All tests were two‐tailed, with a *p* < 0.05 considered statistically significant.

## 3 Results

A total of 22 968 RAMP‐DM participants and 34 558 usual care patients with doctor‐diagnosed non‐complicated T2DM managed in public primary care between 1 August 2009 and 30 September 2010 were identified. Before propensity score matching, usual care patients were generally older, more likely to receive public assistance, had higher HbA1c levels (7.4% vs. 7.2%), longer diabetes duration, higher Charlson Comorbidity Index and lower use of oral anti‐diabetic drugs or lipid‐lowering agents than RAMP‐DM participants (Table S2). Data completeness exceeded 95% for most baseline variables and was generally higher among RAMP‐DM participants due to the comprehensive assessment conducted at enrolment (Table S3). After propensity score matching, 36 746 patients were included (18 373 in each group). Baseline characteristics were well balanced between groups. The mean (SD) age of the matched cohort was 64.7 (12.2) years, mean HbA1c was 7.3 (1.4)% and mean diabetes duration was 7.4 (6.9) years (Table 1).

**TABLE 1:** Baseline characteristics of RAMP‐DM participants and usual care patients after multiple imputation and one‐to‐one propensity score matching.

	Total (*N* = 36 746)	RAMP‐DM participants (*N* = 18 373)	Usual care patients (*N* = 18 373)	ASMD
Socio‐demographics				
Female	52.7% (19 371)	52.9% (9717)	52.5% (9654)	0.007
Age, year	64.7 ± 12.2	64.8 ± 11.0	64.5 ± 13.3	0.028
Current smoker	11.5% (4222)	11.4% (2093)	11.6% (2129)	0.006
On public assistance	11.6% (4254)	12.0% (2197)	11.2% (2057)	0.024
Clinical parameters				
Haemoglobin A1c, %	7.3 ± 1.4	7.3 ± 1.3	7.3 ± 1.4	0.001
Systolic blood pressure, mmHg	136.7 ± 17.4	136.6 ± 16.6	136.7 ± 18.2	0.003
Diastolic blood pressure, mmHg	75.3 ± 10.4	75.2 ± 10.0	75.3 ± 10.8	0.012
LDL‐C, mmol/L	3.1 ± 0.9	3.1 ± 0.8	3.1 ± 0.9	0.001
TC/HDL‐C ratio	4.3 ± 1.4	4.3 ± 1.3	4.3 ± 1.5	0.005
Triglyceride, mmol/L	1.6 ± 1.1	1.6 ± 1.1	1.6 ± 1.1	0.005
Body mass index, kg/m^2^	25.5 ± 4.4	25.5 ± 3.9	25.5 ± 4.9	0.005
eGFR, mL/min/1.73 m^2^	102.1 ± 32.1	102.0 ± 26.2	102.1 ± 37.1	0.001
Urine ACR, mg/mmol	9.6 ± 41.0	8.7 ± 41.1	10.5 ± 46.8	0.041
Clinical characteristics				
Duration of diabetes, year	7.4 ± 6.9	7.4 ± 6.6	7.5 ± 7.1	0.011
Charlson Comorbidity Index^†^	4.2 ± 1.5	4.2 ± 1.4	4.2 ± 1.6	0.020
Hypertension	72.1% (26 481)	71.9% (13 207)	72.2% (13 274)	0.008
Use of insulin	2.9% (1074)	3.0% (547)	2.9% (527)	0.006
Use of oral anti‐diabetic drugs	87.8% (32 248)	87.3% (16 039)	88.2% (16 209)	0.028
Use of anti‐hypertensive drugs	77.5% (28 468)	77.4% (14 226)	77.5% (14 242)	0.002
Use of lipid‐lowering agents	18.6% (6818)	18.6% (3412)	18.5% (3406)	0.001

*Note:* Data are presented as mean ± SD or proportion (*n*).

Abbreviations: ACR, albumin to creatinine ratio; eGFR, estimated glomerular filtration rate; HDL‐C, high‐density lipoprotein cholesterol; LDL‐C, low‐density lipoprotein cholesterol; RAMP‐DM, Risk Assessment and Management Programme ‐ Diabetes Mellitus; TC, total cholesterol.

^†^ The calculation of Charlson Comorbidity Index does not include acquired immune deficiency syndrome (AIDS).

### 3.1 Clinical Effectiveness of RAMP‐DM

Clinical effectiveness estimates were derived from our previously published 10‐year effectiveness evaluation of RAMP‐DM [18]. Over a median follow‐up of 9.3 years, 4952 diabetes‐related complications occurred among RAMP‐DM participants compared with 7166 among patients receiving usual care only over a median follow‐up of 7.1 years (Table S4).

Participants in RAMP‐DM were associated with an ARR of 12.1% for any diabetes‐related complications and 16.2% for all‐cause mortality. Corresponding NNT were 8 and 6, respectively. Annualised incidence rates of adverse outcomes are presented in Table S5. Differences in the incidence of any diabetes‐related complications emerged during the first year of follow‐up and were maintained through year eight, although annual CVD incidence became similar between groups by year nine.

### 3.2 Ten‐Year RAMP‐DM Programme Costs

#### 3.2.1 Fixed Set‐Up Cost

At the Head Office level, a total of US$1.30 million was expended during the first five‐year period, serving 153 344 participants and corresponding to US$8.5 per participant. No additional Head Office set‐up costs were reported during the subsequent 5 years.

At the cluster level, the average cost per participant during the first 5 years was US$1.3 for staff training, US$5.9 for equipment procurement and US$0.2 for other one‐off expenses. Corresponding costs during the second five‐year period were US$5.4, US$5.4 and US$1.1, respectively. Total fixed set‐up costs averaged US$15.9 per participant during the initial implementation phase and US$11.9 during the expansion phase. The overall average fixed set‐up cost was US$13.9 per participant over 10 years. Weighting costs by participant numbers within each phase produced a similar estimate (US$13.2). Detailed cost breakdowns are provided in Table S6.

#### 3.2.2 Recurrent Costs

Mean unit costs for the initial assessment, nurse intervention and doctor intervention were US$31.5 (range: US$24.3–40.7), US$21.9 (range: US$9.3–47.7) and US$29.6 (range: US$15.7–40.4), respectively (Table S7). Annual administrative expenditures at the Head Office totalled US$119 744 for 272 603 participants, equivalent to US$0.4 per participant per year. At the clinic level, mean administrative costs were US$6.9 per participant per year (range: US$0–21.2) (Table S7).

The total programme cost per participant consisted of fixed set‐up costs, intervention costs based on service utilisation and administrative costs. The cumulative 10‐year programme cost was US$329 per participant (Table 2).

**TABLE 2:** Direct medical costs associated with the RAMP‐DM over 10 years.

	Costs (US$)	
	Mean	Range
Fixed set‐up costs^b^	13.9	11.8–55.5
Administrative and recurring expenses		
Head office level (US$0.44/year)	4.4	NA
Clinic level (US$6.94/year)	69.4	0.0–212.4
Sub‐total	73.8	4.4–216.7

RAMP‐DM services costs								
	Intake assessment^a^		Nurse intervention^a^		AC intervention^a^		Costs (US$)	
	Average no. of intervention	Mean cost per patient (US$)	Average no. of intervention	Mean cost per patient (US$)	Average no. of intervention	Mean cost per patient (US$)	Mean	Range
1st year (*n* = 18 219)	1.04	32.8	0.54	11.9	0.34	10.0	54.8	35.2–80.0
2nd year (*n* = 17 996)	0.51	16.2	0.20	4.5	0.21	6.2	26.9	17.6–38.3
3rd year (*n* = 17 730)	0.43	13.5	0.22	4.8	0.24	7.0	25.3	15.8–36.4
4th year (*n* = 17 436)	0.46	14.7	0.17	3.7	0.19	5.7	24.0	15.5–33.6
5th year (*n* = 17 115)	0.33	10.5	0.18	4.0	0.18	5.3	19.8	12.4–28.9
6th year (*n* = 16 730)	0.36	11.3	0.19	4.2	0.18	5.3	20.8	13.4–31.0
7th year (*n* = 16 289)	0.29	9.2	0.19	4.3	0.18	5.4	18.9	11.7–28.1
8th year (*n* = 15 826)	0.28	8.8	0.19	4.1	0.18	5.4	18.3	11.3–27.2
9th year (*n* = 15 110)	0.27	8.6	0.17	3.8	0.16	4.9	17.3	11.1–26.8
10th year (*n* = 3003)	0.24	7.5	0.16	3.6	0.15	4.4	15.5	10.6–27.0
Sub‐total							241.5	154.6–357.7
Total programme cost							329.3	240.0–486.7

*Note:* Only RAMP‐DM participants with full 12‐month follow‐up in each year were included.

Abbreviation: RAMP‐DM, Risk Assessment and Management Programme—Diabetes Mellitus.

^a^ The unit cost of assessment, nurse intervention and associate consultation intervention were US$31.5, US$21.9 and US$29.6, respectively.

^b^ Fixed set‐up cost was assumed to be incurred in the first year.

### 3.3 Direct Medical Costs

Annual public healthcare utilisation rates are summarised in Tables S8 and S9. Compared with patients receiving usual care only, RAMP‐DM participants had significantly lower utilisation of overnight hospitalisations (IRR = 0.50, 95% CI: 0.49–0.52), day hospitalisations (IRR = 0.64, 95% CI: 0.61–0.67), Accident and Emergency Department visits (IRR = 0.63, 95% CI: 0.62–0.65) and Specialist Outpatient Clinic attendances (IRR = 0.72, 95% CI: 0.70–0.73). However, utilisation of GOPCs were higher among RAMP‐DM participants (IRR = 1.16, 95% CI: 1.14–1.17).

Annual direct public medical costs associated with healthcare utilisation are shown in Figure 1, with detailed estimated presented in Table S10. Throughout follow‐up, RAMP‐DM participants had lower public medical costs associated with health services than usual care participants. Annual cost savings ranged from US$543 in year 10 to US$1695 in year 2.

**FIGURE 1:**
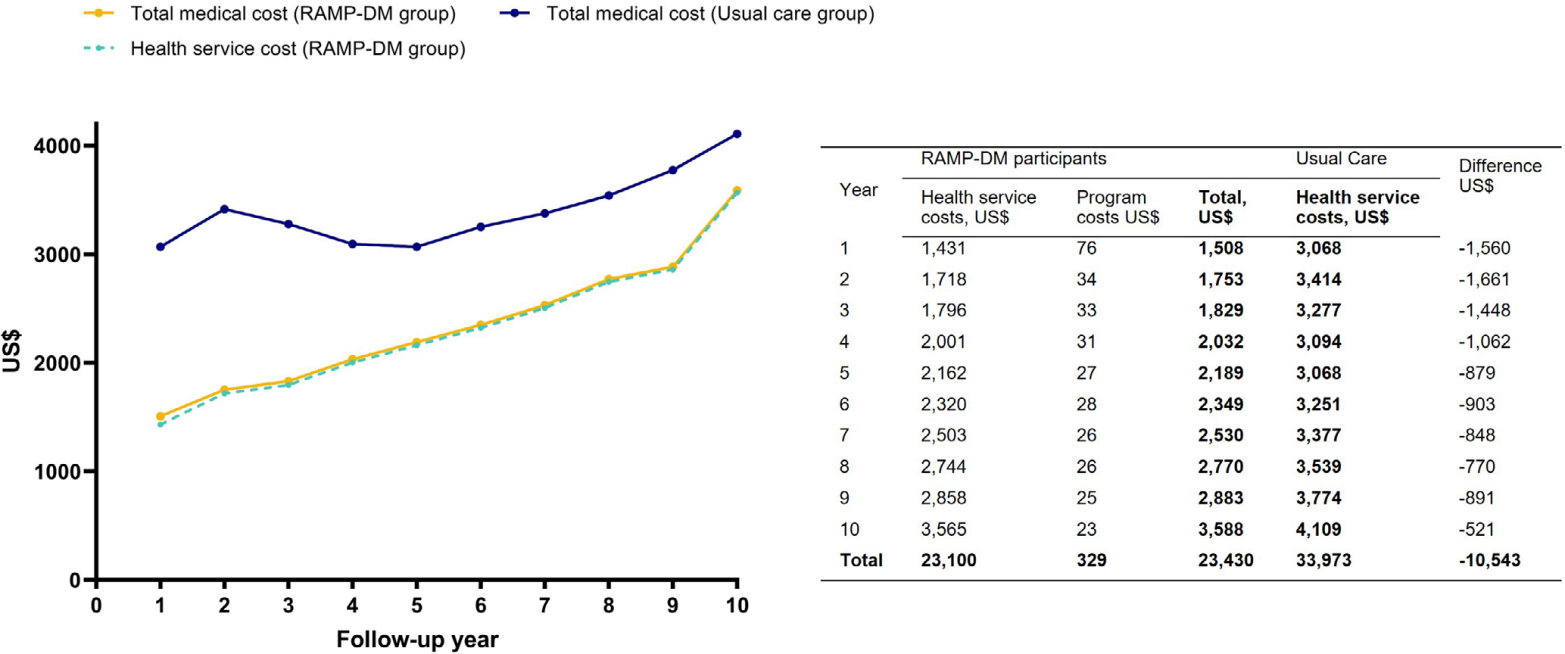
Trends in direct medical costs between RAMP‐DM participants and usual care patients across follow‐up years. RAMP‐DM, Risk Assessment and Management Programme—Diabetes Mellitus; Total medical cost in RAMP‐DM group was the sum of health service cost and RAMP‐DM cost. Total medical cost in usual care group was health service cost.

### 3.4 Ten‐Year Incremental Costs and Clinical Consequences of RAMP‐DM

Over 10 years, total direct medical costs were significantly lower among RAMP‐DM participants (US$23 100) than patients receiving usual care only (US$33 973). After inclusion of programme costs (US$329), participation in RAMP‐DM was associated with net savings of US$10 543 per patient (Figure 1).

Because RAMP‐DM was associated with both lower costs and better clinical outcomes, it was considered economically dominant relative to usual care. The incremental costs and clinical consequences of RAMP‐DM are summarised in Table 3. Using programme cost as an implementation cost metric, the incremental programme cost was US$2733 per diabetes‐related complication avoided and US$2033 per all‐cause mortality avoided. RAMP‐DM remained cost‐saving when annual discount rates of 3% and 5% were applied, with net savings of US$9231 and US$8498, respectively (Tables S11 and S12). When RAMP‐DM recurrent costs were varied by ±10% and ±20%, net savings ranged from US$10 480 to US$10 606. Similarly, one‐way sensitivity analyses demonstrated that uncertainties in model parameters did not affect the conclusion (Figure S1).

**TABLE 3:** Ten‐year cost‐effectiveness of RAMP‐DM.

	Incremental cost		Incremental effectiveness	Incremental cost‐effectiveness ratio	
	(1)	(2)		(1)	(2)
	RAMP‐DM cost, US$ (95% CI^a^)	Total public direct medical cost, US$ (95% CI^a^)	Event prevented per 100 patients (95% CI)^*^	Programme cost per event prevented, US$ (95% CI^a^)	Total public direct medical cost per event prevented, US$
Any complication	329 (325, 333)	−10 543 (−11 691, −9434)	12.1 (11.1, 13.0)	2733 (2531, 2967)	Cost‐saving
CVD	329 (325, 333)	−10 543 (−11 691, −9434)	11.9 (11.0, 12.8)	2764 (2568, 2991)	Cost‐saving
CHD	329 (325, 333)	−10 543 (−11 691, −9434)	6.8 (6.2, 7.5)	4825 (4380, 5348)	Cost‐saving
Heart failure	329 (325, 333)	−10 543 (−11 691, −9434)	4.4 (3.9, 4.9)	7478 (6658, 8512)	Cost‐saving
Stroke	329 (325, 333)	−10 543 (−11 691, −9434)	5.1 (4.5,5.8)	6409 (5654, 7393)	Cost‐saving
PVD	329 (325, 333)	−10 543 (−11 691, −9434)	0.9 (0.7, 1.2)	35 175 (27 506, 49 080)	Cost‐saving
Any microvascular complication	329 (325, 333)	−10 543 (−11 691, −9434)	3.3 (2.6, 4.0)	9951 (8284, 12 379)	Cost‐saving
NPDR	329 (325, 333)	−10 543 (−11 691, −9434)	Not effective		
STDR	329 (325, 333)	−10 543 (−11 691, −9434)	1.0 (0.7, 1.3)	33 426 (26 005, 45 990)	Cost‐saving
Diabetic neuropathy	329 (325, 333)	−10 543 (−11 691, −9434)	0.7 (0.4,0.9)	48 791 (35 877, 76 017)	Cost‐saving
ESRD	329 (325, 333)	−10 543 (−11 691, −9434)	3.2 (2.7, 3.6)	10 413 (9033, 12 266)	Cost‐saving
All‐cause mortality	329 (325, 333)	−10 543 (−11 691, −9434)	16.2 (15.3, 17.1)	2033 (1931, 2148)	Cost‐saving

Abbreviations: CHD, coronary heart disease; CI, confidence interval; CVD, cardiovascular diseases; ESRD, end‐stage renal disease; NNT, number needed to treat; NPDR, non‐proliferative diabetic retinopathy; PVD, peripheral vascular disease; RAMP‐DM, Risk Assessment and Management Programme—Diabetes Mellitus; STDR, sight‐threatening diabetic retinopathy.

^a^ The 95% CI was constructed using bootstrapping with a simulation of 10 000 patients.

* Results for event prevented were taken from our previous study on the 10‐year effectiveness of the RAMP‐DM: [18].

## 4 Discussion

Using territory‐wide real‐world data, this study found that participation in RAMP‐DM was associated with substantially lower direct public medical costs and better clinical outcomes than usual care over 10 years. After accounting for programme costs, RAMP‐DM was associated with net saving of US$10 543 (36.7% relatively lower) per participant. The incremental programme cost was US$2733 per complication avoided. Because costs were lower and outcomes were better, RAMP‐DM was economically dominant form the public healthcare provider perspective.

### 4.1 Long‐Term Costs and Clinical Outcomes

RAMP‐DM demonstrated favourable long‐term economic and clinical outcomes. Over 10 years, participation was associated with annual savings ranging from US$543 to US$1695 in terms of health service utilization, and cumulative savings of US$10 543 per participant. The programme cost was modest, with an incremental programme cost of US$2733 per diabetes‐related complication avoided and US$2033 per all‐cause death avoided. Compared with the previously reported five‐year savings of US$7294 [16], annual savings appeared to diminish over time. This pattern is consistent with the progressive nature of diabetes, where age‐related comorbidities and complications increasingly contribute to healthcare utilisation and mortality despite earlier risk factor modification [18]. Nonetheless, RAMP‐DM remained associated with meaningful reductions in both adverse outcomes and healthcare expenditures throughout the study period.

The cumulative 10‐year programme cost of US$329 per participant was approximately double the previously reported five‐year estimate [16], with the majority of costs attributable to ongoing intervention services. Despite this increase, the average annual programme cost (US$32.9) remained substantially lower than the cost of a single GOPC consultation in Hong Kong (US$57.1) [26] and lower than intervention costs reported in the ADDITION‐UK (~US$1350) [23] and the US Diabetes Prevention Programme (DPP) over 10 years (US$2281–4572) [32]. This relatively low cost likely reflects the integration of RAMP‐DM within existing public primary care infrastructure rather than implementation as a standalone service.

Task‐shifting from physicians to nurses for risk assessments, stratification and lifestyle counselling may have contributed to programme efficiency. On average, RAMP‐DM participants received 2–3 nurse‐led interventions over 10 years in addition to ongoing routine primary care. The value of RAMP‐DM therefore likely attributed to early risk identification, individualised management, timely referral and ongoing monitoring embedded within routine care pathways.

Participation in RAMP‐DM was associated with significantly lower utilisation of hospitalisations, Accident & Emergency department visits and specialist outpatient services. These findings suggest a shift from reactive management of complications towards more proactive and preventive care. Increased GOPC utilisation among RAMP‐DM participants likely reflects greater engagement in regular monitoring. However, differences in healthcare utilisation should not be attributed solely to improved disease control and may also reflect differences in patient engagement, health‐seeking behaviour, follow‐up intensity or other unmeasured factors.

### 4.2 Comparison With Other Studies Evaluating Economic Outcomes of Multidisciplinary Interventions

Our findings are consistent with previous studies supporting the economic value of multidisciplinary diabetes management. Systematic reviews have reported that Chronic Care Model‐based interventions incorporating care coordination, patient education and nurse or pharmacist involvement range from cost‐saving to cost‐effective [33]. Similar economic benefits have been reported for nurse‐led care to be a dominant strategy in 74% of simulations compared to usual care over 2 years of follow‐up [34]. The Diabetes Intense Medical Management ‘tune up’ clinic with endocrinologist‐pharmacist care estimated $5287 (−86.0%) lower 3‐year projected medical costs compared to usual care [35]. High‐levels of nurse involvement in providing clinical‐based activities were associated with lower mean lifetime costs (−AU$8738, 14.3%) and a greater QALY gain (0.3; 95% CI 0.2–0.4) [36]. Multifactorial intervention with cardiovascular risk management and early complications treatment was associated with CHF 7313 (10.7%) savings in medical costs per patient over the lifetime [37]. Unlike these studies, the present study used observed 10‐year clinical outcomes, healthcare utilisation and programme costs from real‐world practice. Although differences in healthcare financing, service delivery and costing methods limit direct comparisons between studies, the present findings provide robust real‐world evidence supporting structured interventions that integrate practice change, behavioural modification and patient education for managing T2DM [33, 38].

### 4.3 Clinical Implications

This study provides empirical evidence that a structured multidisciplinary diabetes management programme may generate sustained clinical benefits while reducing healthcare expenditure from the public healthcare provider perspective. The findings demonstrated that structured, protocol‐driven care, proactive monitoring and multidisciplinary management can be effectively integrated into routine primary care without substantial additional cost.

Although transferability will depend on local workforce structure, financing, healthcare infrastructure and quality of usual care, RAMP‐DM highlights how primary care teams can be empowered to manage chronic diseases more effectively while reducing reliance on specialist and acute services. Risk stratification allows resources to be allocated according to clinical need, potentially improving both patient outcomes and system efficiency.

### 4.4 Strengths and Limitations

This study has several strengths. First, it included a comprehensive territory‐wide costing evaluation of the RAMP‐DM, enhancing the reliability and representativeness of the findings. Second, the large, population‐based cohort and 10‐year follow‐up period strengthen the robustness of the findings. Third, the use of observed incidences of diabetes‐related complications, mortality and healthcare utilisation provided a realistic assessment of programme performance in real‐world clinical practice.

Several limitations should also be considered. First, the analysis was limited to patients managed within the public healthcare system and did not capture costs incurred in the private sector. However, approximately 90% of patients with T2DM in Hong Kong receive care within the public sector [39] and private‐sector expenditures account for approximately 10% of total healthcare expenditures [16, 40]. Second, staff salaries and unit costs were based on 2019 data and applied across the evaluation period. Changes in staffing patterns, service delivery and programme efficiency may have changed over time and the overall programme cost may have been overestimated for earlier years due to inflation. Third, no separate institutional overhead costs were allocated because RAMP‐DM was implemented within existing healthcare infrastructure and capital expenditures were treated as fixed set‐up costs rather than formally annualised. Although this may have influenced cost estimates, the relatively small programme cost compared with the substantial reduction in total direct medical costs makes it unlikely to alter the overall conclusion. Fourth, the primary analysis presented undiscounted costs and outcomes to reflect observed programme performance. However, sensitivity analyses applying discount rates of 3% and 5% produced similar conclusions. Fifth, as an observational study, the analysis remains susceptible residual confounding and selection bias despite propensity‐score matching and imputation for missing baseline variables. Differences in outcomes may partly reflect unmeasured factors including potential selection bias, health‐seeking behaviour, patient engagement, or access to services not captured in administrative data. Sixth, usual care may have evolved during the study period because of broader guidelines implementation. However, any contamination would be expected to reduce differences between groups and may therefore have led to a conservative estimate of programme effectiveness and cost savings. Seventh, the evaluation adopted a healthcare provider perspective and did not include societal costs including productivity loss, caregiver burden, transport costs, or patient out‐of‐pocket expenses. Consequently, the full economic impact of the programme may have been underestimated. Eighth, quality‐adjusted life years could not be estimated because utility‐based quality‐of‐life data were unavailable. The use of natural‐unit outcomes improves transparency but limits direct comparison with conventional cost‐utility analyses. Finally, Hong Kong's heavily subsidised healthcare system may limit the generalisability of these findings to healthcare systems with different financing and organisational structures.

## 5 Conclusions

In this population‐based cohort study, participation in the multidisciplinary, protocol‐driven, risk‐stratified RAMP‐DM was associated with lower 10‐year direct public medical costs and better clinical outcomes. From the public healthcare provider perspective, RAMP‐DM was economically dominant. These findings support continued investment in structured multidisciplinary diabetes management within primary care. Future evaluations incorporating quality‐adjusted life years and societal costs may provide a more comprehensive assessment of the long‐term value of the programme.

## Author Contributions

E.H.M.T. and I.L.M. contributed to the literature review, study design, statistical analysis and writing of the manuscript. E.T.Y.T., J.Y.C., W.Y.C., C.K.H.W., E.Y.F.W. and C.L.K.L. contributed to the study design, acquisition of data and provided expert advice. D.V.K.C., W.W.S.T. and T.K.H.H. provided expert advice and critical input in clinical aspects. All authors reviewed and edited the manuscript and contributed to the interpretation of the results. E.Y.F.W. and C.L.K.L. are the guarantors of this work and, as such, had full access to all the data in the study and take responsibility for the integrity of the data and accuracy of the data analysis.

## Funding

This study was funded by the Health and Medical Research Fund, the Health Bureau, the Government of the Hong Kong Special Administrative Region (Reference number: CFS‐HKU4). The funding source did not have any role, including study design, collection, analysis and interpretation of data, report writing and decision to submit the manuscript for publication, in the current study.

## Ethics Statement

Ethics approval was granted by the Institutional Review Board of The University of Hong Kong/Hospital Authority Hong Kong West Cluster (reference no. UW 19‐329). Informed consent from patients was not required for the use of de‐identified data in this prospective cohort analysis.

## Conflicts of Interest

E.Y.F.W. has received research grants from the Health Bureau, the Hong Kong Research Grants Council, Narcotics Division, Security Bureau, Social Welfare Department, Labour and Welfare Bureau of the Government of the Hong Kong SAR and National Natural Science Foundation of China; serves as a member of Core Team for Expert Group on Drug Registration and a member for Expert Group on Pharmacovigilance of Pharmacy and Poisons Board. These are outside the submitted work. C.L.K.L. has received research grants from the Food and Health Bureau of the Government of the Hong Kong SAR, the Hong Kong Research Grant Council, the Hong Kong College of Family Physicians and Kerry Group Kuok Foundation, outside the submitted work. E.T.Y.T. has received research grants from Kerry Group Kuok Foundation (Hong Kong) Limited, outside the submitted work. The remaining authors declare no conflicts of interest.

## Supporting information


**Table S1:** Unit cost of healthcare service of the Hospital Authority.
**Table S2:** Baseline characteristics of RAMP‐DM participants and usual care patients before propensity score matching.
**Table S3:** Data completion rate of baseline characteristics.
**Table S4:** Incidence of CVD, microvascular complication and all‐cause mortality between RAMP‐DM participants and usual care patients.
**Table S5:** Annualised incidence of CVD, microvascular complication and all‐cause mortality between RAMP‐DM participants and usual care patients.
**Table S6:** Fixed set‐up cost of RAMP‐DM by Hospital Authority clusters.
**Table S7:** Recurrent services and administrative costs of RAMP‐DM by Hospital Authority clusters.
**Table S8:** Frequency of attending healthcare service between RAMP‐DM participants and usual care patients by observation years.
**Table S9:** Negative binomial regression models on public healthcare service uses between RAMP‐DM participants and usual care patients.
**Table S10:** Public direct medical cost incurred by healthcare service utilisation between RAMP‐DM participants and usual care patients by observation years.
**Table S11:** Sensitivity analysis of 10‐year direct medical costs under varying discounted rates and RAMP‐DM recurrent costs.
**Table S12:** Sensitivity analysis of 10‐year cost‐effectiveness of RAMP‐DM under varying discounted rates and RAMP‐DM recurrent costs.
**Figure S1:** Tornado diagram of one‐way sensitivity analyses on factors affecting direct medical costs.

**Data S1:** Supporting Information.

## Data Availability

The data used in this study are not publicly available. Restrictions of data access and the licence to use the data are applied.
