# Supplementary material for: Ten‐Year Cost‐Effectiveness of the Risk Assessment and Management Programme—Diabetes Mellitus (RAMP‐DM) Relative to Usual Care in Hong Kong Public Primary Care: A Population‐Based Cohort Study
Source: Diabetes Obes Metab. 2026 Aug 23;28(11):10379–89. doi: 10.1111/dom.71242 (PMC13646341; doi:10.1111/dom.71242)
Supplement: Supplementary file 2 [file DOM-28-10379-s002.docx]

**Supplemental Materials**

**Ten-year cost-effectiveness of the Risk Assessment and Management Programme - Diabetes Mellitus (RAMP-DM) relative to usual care in Hong Kong public primary care: a population-based cohort study**

Eric Ho Man Tang, Ivy Lynn Mak, Emily Tsui Yee Tse, Esther Yee Tak Yu, Julie Yun Chen, Weng Yee Chin, David Vai Kiong Chao, Wendy Wing Sze Tsui, Tony King Hang Ha, Carlos King Ho Wong, Cindy Lo Kuen Lam, Eric Yuk Fai Wan

| **Description** | **Page** |
| --- | --- |
| Table S1. Unit cost of healthcare service of the Hospital Authority | 3 |
| Table S2. Baseline characteristics of RAMP-DM participants and usual care patients before propensity score matching | 4 |
| Table S3. Data completion rate of baseline characteristics | 5 |
| Table S4. Incidence of CVD, microvascular complication and all-cause mortality between RAMP-DM participants and usual care patients | 6 |
| Table S5. Annualized incidence of CVD, microvascular complication and all-cause mortality between RAMP-DM participants and usual care patients | 7-8 |
| Table S6. Fixed set-up cost of RAMP-DM by Hospital Authority clusters | 9 |
| Table S7. Recurrent services and administrative costs of RAMP-DM by Hospital Authority clusters | 10 |
| Table S8. Frequency of attending healthcare service between RAMP-DM participants and usual care patients by observation years | 11 |
| Table S9. Negative binomial regression models on public healthcare service uses between RAMP-DM participants and usual care patients | 12 |
| Table S10. Public direct medical cost incurred by healthcare service utilization between RAMP-DM participants and usual care patients by observation years | 13-14 |
| Table S11. Sensitivity analysis of ten-year direct medical costs under varying discounted rates and RAMP-DM recurrent costs | 15-17 |
| Table S12. Sensitivity analysis of ten-year cost-effectiveness of RAMP-DM under varying discounted rates and RAMP-DM recurrent costs | 18-20 |
| Figure S1. Tornado diagram of one-way sensitivity analyses on factors affecting direct medical costs | 21 |

# Table S1. Unit cost of healthcare service of the Hospital Authority

| **Healthcare services** | **Unit cost (HK$)** | **Unit cost (US$^a^)** |
| --- | --- | --- |
| Overnight hospitalization, per night |  |  |
| Stay in general ward | 5,100 | 654 |
| Stay in intensive care unit / cardiac care unit | 24,400 | 3,128 |
| Stay in high-dependency unit | 13,650 | 1,750 |
| Day hospitalization, per visit | 1,610 | 206 |
| Accident and Emergency department, per visit | 1,230 | 158 |
| Specialist outpatient clinic, per visit | 1,190 | 153 |
| General outpatient clinic, per visit | 445 | 57 |

Data source: 2017-23 Hong Kong Special Administrative Region Government Gazette and Hospital Authority Ordinance (chapter 113): Public charges – Non-eligible persons.

^a^An exchange rate US$1 = HK$7.8 was used

# Table S2. Baseline characteristics of RAMP-DM participants and usual care patients before propensity score matching

|  | Total (*N* = 57,526) | RAMP-DM participants (*N* = 22,968) | Usual care patients (*N* = 34,558) | ASMD |
| --- | --- | --- | --- | --- |
| **Socio-demographics** |  |  |  |  |
| Female | 52.7% (30,307) | 52.7% (12,102) | 52.7% (18,205) | 0.000 |
| Age, year | 65.4±12.5 | 63.7±10.9 | 66.5±13.4 | 0.227* |
| Current smoker | 11.6% (6,664) | 11.2% (2,572) | 11.8% (4,092) | 0.020 |
| On public assistance | 14.4% (8,307) | 10.3% (2,369) | 17.2% (5,938) | 0.200* |
|  |  |  |  |  |
| **Clinical parameters** |  |  |  |  |
| Haemoglobin A1c, % | 7.3±1.5 | 7.2±1.3 | 7.4±1.7 | 0.129* |
| Systolic blood pressure, mmHg | 136.5±17.9 | 136.9±16.6 | 136.2±18.6 | 0.037 |
| Diastolic blood pressure, mmHg | 74.8±10.6 | 75.9±10.0 | 74.1±11.0 | 0.166* |
| LDL-C, mmol/L | 3.0±0.9 | 3.1±0.8 | 3.0±0.9 | 0.083 |
| TC/HDL-C ratio | 4.3±1.5 | 4.3±1.3 | 4.3±1.6 | 0.028 |
| Triglyceride, mmol/L | 1.6±1.1 | 1.6±1.1 | 1.6±1.2 | 0.011 |
| Body mass index, kg/m^2^ | 25.4±4.4 | 25.5±3.9 | 25.2±4.6 | 0.072 |
| eGFR, mL/min/1.73m^2^ | 100.4±31.2 | 104.5±26.8 | 97.6±33.6 | 0.223* |
| Urine ACR, mg/mmol | 12.9±74.4 | 7.7±36.3 | 16.4±87.5 | 0.120* |
|  |  |  |  |  |
| **Clinical characteristics** |  |  |  |  |
| Duration of diabetes, year | 7.8±7.8 | 7.0±6.3 | 8.4±8.6 | 0.184* |
| Charlson Comorbidity Index^†^ | 4.3±1.6 | 4.1±1.4 | 4.5±1.7 | 0.268* |
| Hypertension | 70.2% (40,402) | 72.5% (16,661) | 68.7% (23,741) | 0.084 |
| Use of insulin | 6.8% (3,913) | 2.4% (555) | 9.7% (3,358) | 0.309* |
| Use of oral anti-diabetic drugs | 84.8% (48,759) | 89.7% (20,595) | 81.5% (28,164) | 0.234* |
| Use of anti-hypertensive drugs | 77.4% (44,508) | 77.3% (17,757) | 77.4% (26,751) | 0.002 |
| Use of lipid-lowering agents | 18.5% (10,659) | 21.0% (4,822) | 16.9% (5,837) | 0.105* |

ACR, albumin to creatinine ratio; ASMD, absolute standardized mean difference; eGFR, estimated glomerular filtration rate; HDL-C, high-density lipoprotein cholesterol; LDL-C, low-density lipoprotein cholesterol; RAMP-DM, Risk Assessment and Management Programme – Diabetes Mellitus; TC, total cholesterol

Notes:

Data are presented as mean±SD or proportion (*n*).

* Imbalance between groups by absolute standardized mean difference > 0.1

† The calculation of Charlson Comorbidity Index does not include Acquired Immune Deficiency Syndrome (AIDS).

# Table S3. Data completion rate of baseline characteristics

|  | Total (*N* = 57,526) | RAMP-DM participants (*N* = 22,968) | Usual care patients (*N* = 34,558) |
| --- | --- | --- | --- |
| **Socio-demographics** |  |  |  |
| Gender | 100.0% (57,526) | 100.0% (22,968) | 100.0% (34,558) |
| Age | 100.0% (57,526) | 100.0% (22,968) | 100.0% (34,558) |
| Smoking status | 94.3% (54,267) | 100.0% (22,968) | 90.6% (31,299) |
| On public assistance | 100.0% (57,526) | 100.0% (22,968) | 100.0% (34,558) |
|  |  |  |  |
| **Clinical parameters** |  |  |  |
| Haemoglobin A1c | 97.0% (55,811) | 100.0% (22,958) | 95.1% (32,853) |
| Systolic blood Pressure | 99.3% (57,144) | 100.0% (22,968) | 98.9% (34,176) |
| Diastolic blood Pressure | 99.3% (57,144) | 100.0% (22,968) | 98.9% (34,176) |
| LDL-C | 95.0% (54,662) | 99.9% (22,947) | 91.8% (31,715) |
| TC/HDL-C ratio | 95.1% (54,727) | 100.0% (22,958) | 91.9% (31,769) |
| Triglyceride | 95.2% (54,775) | 100.0% (22,960) | 92.1% (31,815) |
| Body mass index | 89.0% (51,209) | 99.9% (22,947) | 81.8% (28,262) |
| eGFR | 98.6% (56,702) | 100.0% (22,966) | 97.6% (33,736) |
| Urine ACR | 70.6% (40,611) | 94.5% (21,698) | 54.7% (18,913) |
|  |  |  |  |
| **Clinical Characteristics** |  |  |  |
| Duration of diabetes | 76.8% (44,180) | 99.8% (22,913) | 61.5% (21,267) |
| Charlson Comorbidity Index^†^ | 100.0% (57,526) | 100.0% (22,968) | 100.0% (34,558) |
| Hypertension | 100.0% (57,526) | 100.0% (22,968) | 100.0% (34,558) |
| Use of insulin | 100.0% (57,526) | 100.0% (22,968) | 100.0% (34,558) |
| Use of oral anti-diabetic drugs | 100.0% (57,526) | 100.0% (22,968) | 100.0% (34,558) |
| Use of anti-hypertensive drugs | 100.0% (57,526) | 100.0% (22,968) | 100.0% (34,558) |
| Use of lipid-lowering agents | 100.0% (57,526) | 100.0% (22,968) | 100.0% (34,558) |

ACR, albumin to creatinine ratio; eGFR, estimated glomerular filtration rate; HDL-C, high-density lipoprotein cholesterol; LDL-C, low-density lipoprotein cholesterol; RAMP-DM, Risk Assessment and Management Programme – Diabetes Mellitus; TC = total cholesterol

Note:

^†^ The calculation of Charlson Comorbidity Index does not include Acquired Immune Deficiency Syndrome (AIDS).

**Table S4. Incidence of CVD, microvascular complication and all-cause mortality between RAMP-DM participants and usual care patients**

| **Event** | **RAMP-DM (*N* = 18,373)** | | | | | |  | **Usual care (*N* = 18,373)** | | | | | | ARR, %  (95% CI) | NNT  (95% CI) |
| --- | --- | --- | --- | --- | --- | --- | --- | --- | --- | --- | --- | --- | --- | --- | --- |
|  | Cumulative incidence | | Incidence rate  (case/100 person-years) | | Person-years | Median follow-up periods (years) |  | Cumulative incidence | | Incidence rate  (case/100 person-years) | | Person-years | Median follow-up periods (years) |  |  |
|  | Case with event | Rate, % (95% CI) | Estimate | 95% CI^†^ |  |  |  | Case with event | Rate, % (95% CI) | Estimate | 95% CI^†^ |  |  |  |  |
| Any complication | 4,952 | 26.95 (26.31,27.60) | 3.47 | 3.38 – 3.57 | 142,610 | 9.3 |  | 7,166 | 39.00 (38.30,39.71) | 6.32 | 6.17 – 6.46 | 113,469 | 7.1 | 12.1 (11.1,13.0) | 8.3 (7.7,9.0) |
| CVD | 3,815 | 20.76 (20.18,21.36) | 2.57 | 2.49 – 2.65 | 148,470 | 9.3 |  | 6,004 | 32.68 (32.00,33.36) | 5.05 | 4.92 – 5.18 | 118,990 | 8.1 | 11.9 (11.0,12.8) | 8.4 (7.8,9.1) |
| CHD | 1,656 | 9.01 (8.60,9.44) | 1.06 | 1.01 – 1.11 | 156,510 | 9.4 |  | 2,910 | 15.84 (15.31,16.37) | 2.21 | 2.13 – 2.29 | 131,964 | 9.4 | 6.8 (6.2,7.5) | 14.7 (13.3,16.2) |
| Heart failure | 973 | 5.30 (4.98,5.63) | 0.61 | 0.57 – 0.65 | 159,561 | 9.4 |  | 1,782 | 9.70 (9.27,10.14) | 1.28 | 1.22 – 1.34 | 139,200 | 9.8 | 4.4 (3.9,4.9) | 22.7 (20.2,25.9) |
| Stroke | 1,842 | 10.03 (9.59,10.47) | 1.18 | 1.13 – 1.24 | 155,602 | 9.4 |  | 2,786 | 15.16 (14.65,15.69) | 2.08 | 2.00 – 2.16 | 133,971 | 9.6 | 5.1 (4.5,5.8) | 19.5 (17.2,22.4) |
| PVD | 229 | 1.25 (1.09,1.42) | 0.14 | 0.13 – 0.16 | 161,321 | 9.4 |  | 401 | 2.18 (1.98,2.40) | 0.28 | 0.26 – 0.31 | 142,812 | 9.8 | 0.9 (0.7,1.2) | 106.8 (83.2,149.1) |
| Any microvascular complication | 1,867 | 10.16 (9.73,10.61) | 1.21 | 1.15 – 1.26 | 154,668 | 9.3 |  | 2,475 | 13.47 (12.98,13.97) | 1.83 | 1.76 – 1.90 | 135,509 | 9.6 | 3.3 (2.6,4.0) | 30.2 (25.2,37.7) |
| NPDR | 914 | 4.97 (4.66,5.30) | 0.58 | 0.55 – 0.62 | 157.027 | 9.4 |  | 905 | 4.93 (4.62,5.25) | 0.65 | 0.61 – 0.69 | 139,434 | 9.8 | -0.05 (-0.5,0.4) | NA |
| STDR | 236 | 1.28 (1.13,1.46) | 0.15 | 0.13 – 0.17 | 161,064 | 9.4 |  | 417 | 2.27 (2.06,2.50) | 0.29 | 0.27 – 0.32 | 142,547 | 9.8 | 1.0 (0.7,1.3) | 101.5 (79.7,139.8) |
| Diabetic  neuropathy | 204 | 1.11 (0.96,1.27) | 0.13 | 0.11 – 0.15 | 161,295 | 9.4 |  | 328 | 1.79 (1.60,1.99) | 0.23 | 0.21 – 0.26 | 143,105 | 9.8 | 0.7 (0.4,0.9) | 148.2 (108.8,232.2) |
| ESRD | 798 | 4.34 (4.05,4.65) | 0.50 | 0.46 – 0.53 | 160,886 | 9.4 |  | 1,379 | 7.51 (7.13,7.90) | 0.97 | 0.92 – 1.02 | 142,429 | 9.8 | 3.2 (2.7,3.6) | 31.6 (27.4,37.3) |
| All-cause mortality | 2,961 | 16.12 (15.59,16.66) | 1.83 | 1.76 – 1.89 | 162,163 | 9.4 |  | 5,937 | 32.31 (31.64,33.00) | 4.11 | 4.00 – 4.21 | 144,639 | 9.9 | 16.2 (15.3,17.1) | 6.2 (5.9,6.5) |

ARR, absolute risk reduction; CHD, coronary heart disease; CI, confidence interval; CVD, cardiovascular disease; ESRD, end-stage renal disease; NA, not applicable; NNT, number-needed-to-treat; NPDR, non-proliferative diabetic retinopathy; PVD, peripheral vascular disease; RAMP-DM, Risk Assessment and Management Programme – Diabetes Mellitus; STDR, sight-threatening diabetic retinopathy.

Note:
† The 95% confidence intervals for the incidence rates were constructed based on the Poisson distribution.

**Table S5. Annualized incidence of CVD, microvascular complication and all-cause mortality between RAMP-DM participants and usual care patients**

|  | Group | 1-year | 2-year | 3-year | 4-year | 5-year | 6-year | 7-year | 8-year | 9-year | 10-year |
| --- | --- | --- | --- | --- | --- | --- | --- | --- | --- | --- | --- |
| Any complication | RAMP-DM | 4.02% | 3.43% | 3.11% | 3.00% | 2.99% | 3.13% | 3.03% | 3.46% | 3.46% | 4.16% |
|  | Usual care | 9.54% | 8.22% | 5.65% | 5.06% | 4.22% | 4.18% | 4.05% | 3.72% | 4.08% | 4.08% |
|  | P-value | <0.001* | <0.001* | <0.001* | <0.001* | <0.001* | <0.001* | <0.001* | 0.275 | 0.014* | 0.841 |
| CVD | RAMP-DM | 2.46% | 2.40% | 2.34% | 2.25% | 2.36% | 2.47% | 2.38% | 2.55% | 2.92% | 2.97% |
|  | Usual care | 7.67% | 6.74% | 4.59% | 4.12% | 3.41% | 3.38% | 3.31% | 3.06% | 3.30% | 3.19% |
|  | P-value | <0.001* | <0.001* | <0.001* | <0.001* | <0.001* | <0.001* | <0.001* | 0.015* | 0.091 | 0.504 |
| CHD | RAMP-DM | 1.01% | 1.01% | 0.96% | 0.81% | 0.99% | 1.02% | 0.95% | 1.12% | 1.27% | 1.07% |
|  | Usual care | 3.59% | 3.02% | 2.12% | 1.77% | 1.54% | 1.39% | 1.52% | 1.46% | 1.38% | 1.43% |
|  | P-value | <0.001* | <0.001* | <0.001* | <0.001* | <0.001* | 0.003* | <0.001* | 0.013* | 0.471 | 0.087 |
| Heart failure | RAMP-DM | 0.51% | 0.50% | 0.53% | 0.57% | 0.50% | 0.57% | 0.58% | 0.69% | 0.80% | 0.83% |
|  | Usual care | 1.59% | 1.71% | 1.21% | 1.08% | 1.02% | 1.01% | 0.92% | 0.95% | 0.98% | 1.01% |
|  | P-value | <0.001* | <0.001* | <0.001* | <0.001* | <0.001* | <0.001* | <0.001* | 0.011* | 0.103 | 0.323 |
| Stroke | RAMP-DM | 1.14% | 1.10% | 1.12% | 1.09% | 1.16% | 1.06% | 1.06% | 1.21% | 1.22% | 1.27% |
|  | Usual care | 3.18% | 2.78% | 1.90% | 1.70% | 1.51% | 1.65% | 1.48% | 1.41% | 1.48% | 1.18% |
|  | P-value | <0.001* | <0.001* | <0.001* | <0.001* | 0.008* | <0.001* | 0.001* | 0.155 | 0.068 | 0.661 |
| PVD | RAMP-DM | 0.07% | 0.11% | 0.11% | 0.16% | 0.10% | 0.23% | 0.23% | 0.11% | 0.14% | 0.19% |
|  | Usual care | 0.39% | 0.31% | 0.29% | 0.26% | 0.17% | 0.18% | 0.36% | 0.17% | 0.24% | 0.18% |
|  | P-value | <0.001* | <0.001* | <0.001* | 0.060 | 0.102 | 0.380 | 0.024* | 0.134 | 0.055 | 0.906 |
| Any microvascular complication | RAMP-DM | 1.69% | 1.22% | 0.97% | 1.05% | 0.97% | 1.07% | 1.07% | 1.20% | 1.01% | 1.61% |
|  | Usual care | 2.49% | 2.20% | 1.50% | 1.49% | 1.33% | 1.43% | 1.48% | 1.39% | 1.52% | 1.57% |
|  | P-value | <0.001* | <0.001* | <0.001* | <0.001* | 0.002* | 0.005* | 0.002* | 0.140 | <0.001* | 0.881 |
| DPNR | RAMP-DM | 1.32% | 0.82% | 0.54% | 0.42% | 0.42% | 0.38% | 0.39% | 0.46% | 0.30% | 0.37% |
|  | Usual care | 1.38% | 1.04% | 0.62% | 0.56% | 0.32% | 0.44% | 0.30% | 0.34% | 0.37% | 0.28% |
|  | P-value | 0.587 | 0.033* | 0.315 | 0.055 | 0.132 | 0.412 | 0.215 | 0.119 | 0.374 | 0.355 |
| STDR | RAMP-DM | 0.26% | 0.08% | 0.11% | 0.12% | 0.13% | 0.15% | 0.18% | 0.18% | 0.06% | 0.21% |
|  | Usual care | 0.54% | 0.33% | 0.22% | 0.16% | 0.23% | 0.23% | 0.31% | 0.20% | 0.26% | 0.18% |
|  | P-value | <0.001* | <0.001* | 0.011* | 0.343 | 0.039* | 0.108 | 0.027* | 0.695 | <0.001* | 0.680 |
| Neuropathy | RAMP-DM | 0.09% | 0.13% | 0.13% | 0.15% | 0.10% | 0.22% | 0.17% | 0.07% | 0.09% | 0.05% |
|  | Usual care | 0.31% | 0.27% | 0.22% | 0.21% | 0.15% | 0.16% | 0.28% | 0.17% | 0.22% | 0.12% |
|  | P-value | <0.001* | 0.002* | 0.048* | 0.151 | 0.235 | 0.271 | 0.046* | 0.010* | 0.006* | 0.219 |
| ESRD | RAMP-DM | 0.15% | 0.25% | 0.28% | 0.50% | 0.41% | 0.53% | 0.56% | 0.65% | 0.70% | 1.27% |
|  | Usual care | 0.56% | 0.81% | 0.72% | 0.81% | 0.89% | 0.87% | 1.13% | 0.93% | 1.05% | 1.28% |
|  | P-value | <0.001* | <0.001* | <0.001* | <0.001* | <0.001* | <0.001* | <0.001* | 0.006* | 0.001* | 0.961 |
| All-cause mortality | RAMP-DM | 0.82% | 1.07% | 1.25% | 1.42% | 1.60% | 1.96% | 2.33% | 2.24% | 2.83% | 2.95% |
|  | Usual care | 3.94% | 4.65% | 4.15% | 3.56% | 3.47% | 3.37% | 3.51% | 3.62% | 3.73% | 3.74% |
|  | P-value | <0.001* | <0.001* | <0.001* | <0.001* | <0.001* | <0.001* | <0.001* | <0.001* | <0.001* | 0.016* |

CHD, coronary heart disease; CVD, cardiovascular disease; ESRD, end-stage renal disease; PVD, peripheral vascular disease; RAMP-DM, Risk Assessment and Management Programme – Diabetes Mellitus; STDR, sight-threatening diabetic retinopathy.

Note:

* Significant at 0.05 level by Chi-square test.

**Table S6. Fixed set-up cost of RAMP-DM by Hospital Authority clusters**

| **Initial fixed set-up cost (1^st^ to 5^th^ years)** | | | | | | | | |
| --- | --- | --- | --- | --- | --- | --- | --- | --- |
| **Cluster** | **A** | **B** | **C** | **D** | **E** | **F** | **G** | **Average cost  per patient**^c^ **(US$)** |
| Number of patients served | 7,938 | 15,776 | 15,837 | 31,177 | 26,560 | 17,080 | 12,286 | Mean (range) |
| **Head Office** | US$1.30 million / 126,654 _RAMP-DM_ + 26,690 _RAMP-HT_ | | | | | | | **8.5** |
|  |  |  |  |  |  |  |  |  |
| **Cluster (average cost per patient, US$)^†^** | | | | | | | | |
| Training | 2.2 | 1.8 | 0.8 | 0.3 | 0.0^a^ | 0.6 | 1.7 | 1.3 (0.3, 2.2) |
| Equipment | 3.2 | 1.1 | 2.3 | 11.0 | 7.2 | 8.2 | 8.3 | 5.9 (1.1, 11.0) |
| Others^d^ | 0.2 | 0.1 | 0.2 | 0.3 | 0.7 | 0.1 | 0 | 0.2 (0, 0.7) |
| Sub-total^e^ | 5.6 | 3.0 | 3.4 | 11.6 | 8.0 | 9.0 | 10.0 | **7.4 (3.0, 11.6)** |
|  |  |  |  |  |  |  |  |  |
| **Total (US$)**^f^ |  |  |  |  |  |  |  | **15.9 (11.5, 20.0)** |
| **Further fixed set-up cost (6^th^ to 10^th^ years)** | | | | | | | | |
| Number of patients served | 14,898 | 38,104 | 40,135 | 61,804 | 35,531 | 26,372 | 44,106 | Mean (range) |
| **Cluster (average cost per patient, US$)**^b^ | | | | | | | | |
| Training | 21.0 | 1.1 | 6.4 | 0.3 | 0.7 | 4.4 | 3.8 | 5.4 (0.3, 21.0) |
| Equipment | 9.6 | 3.3 | 7.5 | 0.1 | 3.7 | 8.0 | 5.8 | 5.4 (0.1, 9.6) |
| Others^d^ | 4.9 | 0.5 | 1.9 | 0 | 0.6 | 0 | 0 | 1.1 (0, 4.9) |
| **Total (US$)**^e^ | 35.5 | 4.8 | 15.8 | 0.3 | 5.0 | 12.4 | 9.6 | **11.9 (0.3, 35.5)** |
| **Average set-up cost over 10 years** | | | |  |  |  |  | **13.9 (1.7, 27.8)** |

RAMP-DM, Risk Assessment and Management Programme – Diabetes Mellitus

Notes:

^a^ Excluded in the calculation of the average cost as it was assumed that initial training cost would not be zero.

^b^ The average cost per patient for each cluster were calculated by the total costs incurred at individual cluster dividing by the number of patients served in that cluster.

^c^ The overall average cost per patient were the average cost across all clusters.

^d^ Others included costs incurred by purchase of furniture and renovation of venue.

^e^ The sub-total/total cost in each cluster was the sum of training, equipment and other costs at that cluster.

^f^ The total initial fixed set-up cost was the sum of the cost in the Head Office and clusters.

**Table S7. Recurrent services and administrative costs of RAMP-DM by Hospital Authority clusters**

| **Cost items (US$) / Cluster** | **A** | **B** | **C** | **D** | **E** | **F** | **G** | **Average cost (range)** |  |
| --- | --- | --- | --- | --- | --- | --- | --- | --- | --- |
| **RAMP-DM services (Unit cost per attendance, US$)** | | | | | | | | | |
| a) **Assessment** | | | | | | | | | |
| Staff | 27.3 | 28.4 | 29.9 | 20.7 | 35.2 | 38.6 | 25.8 | 29.4 (20.7, 38.6) |  |
| Resources^a^ | 1.3 | 1.3 | 1.8 | 3.6 | 1.6 | 2.1 | 3.1 | 2.1 (1.3, 3.6) |  |
| **Total** | 28.6 | 29.8 | 31.7 | 24.3 | 36.8 | 40.7 | 28.8 | **31.5 (24.3, 40.7)** |  |
|  |  |  |  |  |  |  |  |  |  |
| b) **Nurse intervention** | | | | | | | | | |
| Staff | 11.3 | 18.0 | 22.3 | 29.6 | 46.1 | 13.8 | 9.0 | 21.4 (9.0, 46.1) |  |
| Resources^a^ | 0.3 | 0.4 | 0.2 | 0.7 | 1.6 | 0.01 | 0.4 | 0.5 (0.01, 1.6) |  |
| **Total** | 11.6 | 18.4 | 22.5 | 30.3 | 47.7 | 13.8 | 9.3 | **21.9 (9.3, 47.7)** |  |
|  |  |  |  |  |  |  |  |  |  |
| c) **Associate consultant intervention** | | | | | | | | | |
| Staff | 35.1 | 33.5 | 39.0 | 15.7 | 24.7 | 27.7 | 23.9 | 28.5 (15.7, 39.0) |  |
| Resources^a^ | 0.4 | 0.7 | 1.3 | 0 | 2.3 | 2.2 | 1.2 | 1.1 (0, 2.3) |  |
| **Total** | 35.4 | 34.1 | 40.4 | 15.7 | 27.0 | 29.9 | 25.1 | **29.6 (15.7, 40.4)** |  |
| **Administrative costs per patient served** | | | | |  |  |  |  |  |
| Staff | 0 | 1.3 | 2.6 | 21.2 | 9.8 | 12.3 | 0 | 6.7 (0.0, 21.2) |  |
| Resources | 1.3 | 0 | 0.1 | 0 | 0.1 | 0 | 0 | 0.2 (0.0, 1.3) |  |
| **Total** | 1.3 | 1.3 | 2.7 | 21.2 | 9.9 | 12.3 | 0 | **6.9 (0.0, 21.2)** |  |

RAMP-DM, Risk Assessment and Management Programme – Diabetes Mellitus

Note:

^a^ Resources included bottles, test tubes, lancets, test strips, monofilaments, stationery and pamphlets.

**Table S8. Frequency of healthcare services attendance between RAMP-DM participants and usual care patients by observation years**

| **Attendance of healthcare services, episode (mean±SD)** | **1^st^ year** | | | **2^nd^ year** | | | **3^rd^ year** | | | **4^th^ year** | | | **5^th^ year** | | |
| --- | --- | --- | --- | --- | --- | --- | --- | --- | --- | --- | --- | --- | --- | --- | --- |
|  | RAMP-DM participants (*N*=18,219) | Usual care patients (*N*=17,544) | P-value | RAMP-DM participants (*N*=17,996) | Usual care patients (*N*=16,498) | P-value | RAMP-DM participants (*N*=17,730) | Usual care patients (*N*=15,638) | P-value | RAMP-DM participants (*N*=17,436) | Usual care patients (*N*=14,942) | P-value | RAMP-DM participants (*N*=17,115) | Usual care patients (*N*=14,279) | P-value |
| Overnight hospitalization | 0.18±0.70 | 0.43±1.16 | <0.001* | 0.21±0.74 | 0.47±1.21 | <0.001* | 0.24±0.81 | 0.46±1.22 | <0.001* | 0.27±0.89 | 0.44±1.23 | <0.001* | 0.29±0.91 | 0.45±1.25 | <0.001* |
| Total length of stay, night | 1.10±7.01 | 3.12±12.51 | <0.001* | 1.48±8.75 | 3.65±14.78 | <0.001* | 1.62±9.22 | 3.51±15.15 | <0.001* | 1.91±10.84 | 3.28±14.07 | <0.001* | 2.14±11.98 | 3.27±13.59 | <0.001* |
| General ward, night | 1.09±6.95 | 3.05±12.32 | <0.001* | 1.45±8.61 | 3.57±14.56 | <0.001* | 1.60±9.11 | 3.45±15.01 | <0.001* | 1.88±10.70 | 3.23±13.85 | <0.001* | 2.11±11.83 | 3.22±13.44 | <0.001* |
| Intensive care unit, night | 0.01±0.25 | 0.04±0.88 | <0.001* | 0.02±0.47 | 0.04±0.79 | 0.002* | 0.01±0.30 | 0.03±0.60 | <0.001* | 0.02±0.33 | 0.03±0.75 | 0.034* | 0.02±0.63 | 0.02±0.60 | 0.746 |
| Cardiac care unit, night | 0.01±0.22 | 0.03±0.42 | <0.001* | 0.01±0.28 | 0.03±0.44 | <0.001* | 0.01±0.30 | 0.03±0.41 | <0.001* | 0.01±0.26 | 0.02±0.44 | 0.016* | 0.01±0.17 | 0.02±0.34 | <0.001* |
| High-dependency unit, night | 0.00±0.04 | 0.00±0.10 | 0.001* | 0.00±0.03 | 0.00±0.15 | <0.001* | 0.00±0.03 | 0.00±0.11 | 0.002* | 0.00±0.12 | 0.00±0.04 | 0.556 | 0.00±0.19 | 0.00±0.39 | 0.750 |
| Day hospitalization | 0.11±0.68 | 0.19±0.98 | <0.001* | 0.12±0.76 | 0.21±1.05 | <0.001* | 0.13±0.85 | 0.21±1.27 | <0.001* | 0.15±1.19 | 0.22±1.66 | <0.001* | 0.16±1.46 | 0.23±1.93 | <0.001* |
| A&E department | 0.42±1.09 | 0.65±1.62 | <0.001* | 0.44±1.12 | 0.68±1.65 | <0.001* | 0.46±1.52 | 0.68±1.78 | <0.001* | 0.48±1.26 | 0.64±1.69 | <0.001* | 0.48±1.25 | 0.66±1.96 | <0.001* |
| Specialist outpatient clinic | 1.82±2.98 | 2.43±3.46 | <0.001* | 1.93±3.11 | 3.03±3.90 | <0.001* | 2.08±3.23 | 3.16±3.89 | <0.001* | 2.16±3.25 | 3.19±3.80 | <0.001* | 2.26±3.29 | 3.25±3.77 | <0.001* |
| General outpatient clinic | 5.28±2.37 | 5.84±3.89 | <0.001* | 4.95±2.34 | 4.22±3.80 | <0.001* | 4.59±2.41 | 3.58±3.59 | <0.001* | 4.37±2.56 | 3.32±3.61 | <0.001* | 4.16±2.58 | 3.14±3.47 | <0.001* |

| **Attendance of healthcare services, episode (mean±SD)** | **6^th^ year** | | | **7^th^ year** | | | **8^th^ year** | | | **9^th^ year** | | | **10^th^ year** | | |
| --- | --- | --- | --- | --- | --- | --- | --- | --- | --- | --- | --- | --- | --- | --- | --- |
|  | RAMP-DM participants (*N*=16,730) | Usual care patients (*N*=13,689) | P-value | RAMP-DM participants (*N*=16,289) | Usual care patients (*N*=13,087) | P-value | RAMP-DM participants (*N*=15,826) | Usual care patients (*N*=12,458) | P-value | RAMP-DM participants (*N*=15,110) | Usual care patients (*N*=11,735) | P-value | RAMP-DM participants (*N*=3,003) | Usual care patients (*N*=9,341) | P-value |
| Overnight hospitalization | 0.34±1.05 | 0.49±1.42 | <0.001* | 0.36±1.04 | 0.52±1.41 | <0.001* | 0.41±1.18 | 0.54±1.42 | <0.001* | 0.44±1.27 | 0.57±1.51 | <0.001* | 0.52±1.34 | 0.61±1.61 | 0.003* |
| Total length of stay, night | 2.36±11.09 | 3.52±15.12 | <0.001* | 2.61±12.51 | 3.68±15.24 | <0.001* | 2.92±12.63 | 3.93±15.44 | <0.001* | 3.04±12.20 | 4.25±16.87 | <0.001* | 3.71±13.37 | 4.67±17.90 | 0.002* |
| General ward, night | 2.32±10.88 | 3.48±14.99 | <0.001* | 2.57±12.40 | 3.63±15.09 | <0.001* | 2.88±12.50 | 3.89±15.28 | <0.001* | 3.00±12.07 | 4.20±16.76 | <0.001* | 3.60±12.88 | 4.61±17.78 | <0.001* |
| Intensive care unit, night | 0.02±0.44 | 0.03±0.77 | 0.247 | 0.02±0.39 | 0.03±0.47 | 0.037* | 0.02±0.48 | 0.02±0.43 | 0.821 | 0.02±0.51 | 0.03±0.43 | 0.292 | 0.08±2.23 | 0.03±0.53 | 0.274 |
| Cardiac care unit, night | 0.01±0.27 | 0.02±0.41 | 0.045* | 0.01±0.24 | 0.02±0.69 | 0.076 | 0.01±0.32 | 0.02±0.43 | 0.265 | 0.02±0.48 | 0.02±0.37 | 0.899 | 0.03±0.65 | 0.02±0.43 | 0.636 |
| High-dependency unit, night | 0.00±0.31 | 0.00±0.13 | 0.728 | 0.01±0.68 | 0.00±0.03 | 0.199 | 0.00±0.08 | 0.00±0.26 | 0.174 | 0.00±0.07 | 0.00±0.06 | 0.694 | 0.00±0.13 | 0.00±0.13 | 0.596 |
| Day hospitalization | 0.17±1.17 | 0.25±2.36 | <0.001* | 0.20±1.69 | 0.28±2.60 | 0.003* | 0.22±1.85 | 0.30±2.80 | 0.004* | 0.22±1.98 | 0.30±2.74 | 0.011* | 0.31±2.73 | 0.35±3.33 | 0.476 |
| A&E department | 0.52±1.33 | 0.66±1.59 | <0.001* | 0.52±1.21 | 0.68±1.72 | <0.001* | 0.55±1.33 | 0.68±1.80 | <0.001* | 0.57±1.28 | 0.71±2.03 | <0.001* | 0.66±1.35 | 0.74±1.61 | 0.008* |
| Specialist outpatient clinic | 2.39±3.39 | 3.34±3.75 | <0.001* | 2.49±3.38 | 3.39±3.74 | <0.001* | 2.67±3.48 | 3.52±3.80 | <0.001* | 2.80±3.54 | 3.61±3.77 | <0.001* | 3.33±3.77 | 3.82±3.93 | <0.001* |
| General outpatient clinic | 3.98±2.63 | 3.00±3.86 | <0.001* | 3.76±2.55 | 2.91±3.95 | <0.001* | 3.69±2.61 | 2.82±3.77 | <0.001* | 3.57±2.60 | 2.78±3.55 | <0.001* | 3.62±3.14 | 2.69±3.22 | <0.001* |

A&E, Accident and Emergency; SD, Standard deviation; RAMP-DM, Risk Assessment and Management Programme – Diabetes Mellitus

Notes:

* Significant at 0.05 level by generalized linear model with Poisson family, log link and robust standard errors.

Patients with full 12-month follow-up in each year were included.

**Table S9. Negative binomial regression models on public healthcare service uses between RAMP-DM participants and usual care patients**

|  | **RAMP-DM (vs. usual care only)** | | |
| --- | --- | --- | --- |
|  | IRR | 95% CI | P-value |
| Overnight hospitalization | 0.50 | (0.49,0.52) | <0.001* |
| Day hospitalization | 0.64 | (0.61,0.67) | <0.001* |
| Accident and Emergency department | 0.63 | (0.62,0.65) | <0.001* |
| Specialist outpatient clinic | 0.72 | (0.70,0.73) | <0.001* |
| General outpatient clinic | 1.16 | (1.14,1.17) | <0.001* |

CI, Confidence interval; IRR, Incidence rate ratio; RAMP-DM, Risk Assessment and Management Programme – Diabetes Mellitus

Notes:

The negative binomial regression models used person-time as an offset.

* Significant at 0.05 level by negative binomial regression model.

**Table S10. Public direct medical cost incurred by healthcare service utilization between RAMP-DM participants and usual care patients by observation years**

| Direct medical cost, US$ (mean±SD) | 1^st^ year | | | 2^nd^ year | | | 3^rd^ year | | |
| --- | --- | --- | --- | --- | --- | --- | --- | --- | --- |
|  | RAMP-DM participants  (*N*=18,219) | Usual care patients (*N*=17,544) | P-value | RAMP-DM participants  (*N*=17,996) | Usual care patients (*N*=16,498) | P-value | RAMP-DM participants  (*N*=17,730) | Usual care patients (*N*=15,638) | P-value |
| Overnight hospitalization | 763±4,824 | 2,222±9,058 | <0.001* | 1,047±6,250 | 2,562±10,489 | <0.001* | 1,116±6,399 | 2,440±10,450 | <0.001* |
| Stay in general ward | 710±4,544 | 1,994±8,053 | <0.001* | 950±5,627 | 2,335±9,519 | <0.001* | 1,045±5,959 | 2,256±9,814 | <0.001* |
| Stay in intensive care unit | 32±788 | 126±2,751 | <0.001* | 61±1,455 | 129±2,458 | 0.002* | 40±946 | 100±1,885 | <0.001* |
| Stay in cardiac care unit | 20±686 | 95±1,308 | <0.001* | 35±862 | 90±1,375 | <0.001* | 30±937 | 78±1,275 | <0.001* |
| Stay in high-dependency unit | 1±71 | 6±179 | 0.001* | 1±57 | 8±258 | <0.001* | 1±59 | 6±186 | 0.002* |
| Day hospitalization | 23±139 | 39±202 | <0.001* | 24±156 | 43±217 | <0.001* | 28±175 | 44±261 | <0.001* |
| A&E department | 67±171 | 103±256 | <0.001* | 70±177 | 107±261 | <0.001* | 72±239 | 106±281 | <0.001* |
| Specialist outpatient clinic | 278±454 | 371±527 | <0.001* | 295±474 | 462±595 | <0.001* | 318±493 | 483±594 | <0.001* |
| General outpatient clinic | 301±135 | 333±222 | <0.001* | 283±134 | 241±217 | <0.001* | 262±138 | 204±205 | <0.001* |
| **Total** | **1,431±5,016** | **3,068±9,304** | **<0.001*** | **1,718±6,442** | **3,414±10,707** | **<0.001*** | **1,796±6,601** | **3,277±10,676** | **<0.001*** |
| Direct medical cost, US$ (mean±SD) | 4^th^ year | | | 5^th^ year | | | 6^th^ year | | |
|  | RAMP-DM participants  (*N*=17,436) | Usual care patients (*N*=14,942) | P-value | RAMP-DM participants  (*N*=17,115) | Usual care patients (*N*=14,279) | P-value | RAMP-DM participants  (*N*=16,730) | Usual care patients (*N*=13,689) | P-value |
| Overnight hospitalization | 1,316±7,494 | 2,272±10,026 | <0.001* | 1,472±8,384 | 2,243±9,503 | <0.001* | 1,611±7,840 | 2,415±10,495 | <0.001* |
| Stay in general ward | 1,231±6,998 | 2,114±9,057 | <0.001* | 1,378±7,734 | 2,108±8,788 | <0.001* | 1,520±7,114 | 2,273±9,802 | <0.001* |
| Stay in intensive care unit | 51±1,048 | 95±2,339 | 0.034* | 69±1,985 | 76±1,882 | 0.746 | 58±1,364 | 85±2,393 | 0.247 |
| Stay in cardiac care unit | 31±810 | 62±1,372 | 0.016* | 19±546 | 51±1,060 | <0.001* | 26±833 | 52±1,288 | 0.045* |
| Stay in high-dependency unit | 3±215 | 2±79 | 0.556 | 6±331 | 8±682 | 0.750 | 6±539 | 5±232 | 0.728 |
| Day hospitalization | 30±245 | 45±343 | <0.001* | 32±302 | 47±398 | <0.001* | 35±241 | 52±487 | <0.001* |
| A&E department | 75±199 | 101±266 | <0.001* | 76±197 | 104±309 | <0.001* | 82±210 | 104±250 | <0.001* |
| Specialist outpatient clinic | 330±496 | 487±580 | <0.001* | 345±502 | 495±575 | <0.001* | 365±517 | 510±573 | <0.001* |
| General outpatient clinic | 249±146 | 190±206 | <0.001* | 237±147 | 179±198 | <0.001* | 227±150 | 171±220 | <0.001* |
| **Total** | **2,001±7,700** | **3,094±10,260** | **<0.001*** | **2,162±8,578** | **3,068±9,770** | **<0.001*** | **2,320±8,070** | **3,251±10,740** | **<0.001*** |

A&E, Accident and Emergency; RAMP-DM, Risk Assessment and Management Programme – Diabetes Mellitus; SD, Standard deviation.

Notes:

* Significant at 0.05 level by generalized linear model with Poisson family, log link and robust standard errors.

Patients with full 12-month follow-up in each year were included.

**Table S10 (continued). Public direct medical cost incurred by healthcare service utilization between RAMP-DM participants and usual care patients by observation years**

| Direct medical cost, US$ (mean±SD) | 7^th^ year | | | 8^th^ year | | | 9^th^ year | | |
| --- | --- | --- | --- | --- | --- | --- | --- | --- | --- |
|  | RAMP-DM participants  (*N*=16,289) | Usual care patients (*N*=13,087) | P-value | RAMP-DM participants  (*N*=15,826) | Usual care patients (*N*=12,458) | P-value | RAMP-DM participants  (*N*=15,110) | Usual care patients (*N*=11,735) | P-value |
| Overnight hospitalization | 1,786±8,604 | 2,531±10,610 | <0.001* | 1,994±8,733 | 2,673±10,577 | <0.001* | 268±1,090 | 2,891±11,414 | <0.001* |
| Stay in general ward | 1,683±8,110 | 2,371±9,867 | <0.001* | 1,883±8,172 | 2,543±9,991 | <0.001* | 1,962±7,892 | 2,745±10,957 | <0.001* |
| Stay in intensive care unit | 60±1,233 | 94±1,479 | 0.037* | 69±1,497 | 65±1,346 | 0.821 | 67±1,584 | 86±1,333 | 0.292 |
| Stay in cardiac care unit | 30±745 | 65±2,165 | 0.076 | 41±1,002 | 57±1,343 | 0.265 | 60±1,490 | 58±1,144 | 0.899 |
| Stay in high-dependency unit | 13±1,186 | 1±59 | 0.199 | 2±146 | 8±450 | 0.174 | 3±116 | 2±110 | 0.694 |
| Day hospitalization | 41±349 | 57±537 | 0.003* | 45±382 | 62±578 | 0.004* | 46±409 | 62±565 | 0.011* |
| A&E department | 82±192 | 107±271 | <0.001* | 87±209 | 107±283 | <0.001* | 89±202 | 112±320 | <0.001* |
| Specialist outpatient clinic | 380±515 | 517±571 | <0.001* | 407±530 | 537±580 | <0.001* | 427±541 | 550±575 | <0.001* |
| General outpatient clinic | 215±146 | 166±225 | <0.001* | 210±149 | 161±215 | <0.001* | 204±148 | 159±203 | <0.001* |
| **Total** | **2,503±8,816** | **3,377±10,880** | **<0.001*** | **2,744±8,978** | **3,539±10,865** | **<0.001*** | **2,858±8,781** | **3,774±11,694** | **<0.001*** |
| Direct medical cost, US$ (mean±SD) | 10^th^ year | | |  |  |  |  |  |  |
|  | RAMP-DM participants  (*N*=3,003) | Usual care patients (*N*=9,341) | P-value |  |  |  |  |  |  |
| Overnight hospitalization | 2,684±11,795 | 3,183±12,137 | 0.045* |  |  |  |  |  |  |
| Stay in general ward | 2,352±8,419 | 3,014±11,623 | <0.001* |  |  |  |  |  |  |
| Stay in intensive care unit | 236±6,991 | 95±1,662 | 0.274 |  |  |  |  |  |  |
| Stay in cardiac care unit | 88±2,023 | 69±1,341 | 0.636 |  |  |  |  |  |  |
| Stay in high-dependency unit | 7±226 | 5±222 | 0.596 |  |  |  |  |  |  |
| Day hospitalization | 64±563 | 72±687 | 0.476 |  |  |  |  |  |  |
| A&E department | 104±214 | 117±254 | 0.008* |  |  |  |  |  |  |
| Specialist outpatient clinic | 508±576 | 583±599 | <0.001* |  |  |  |  |  |  |
| General outpatient clinic | 207±179 | 154±184 | <0.001* |  |  |  |  |  |  |
| **Total** | **3,565±12,035** | **4,109±12,425** | **0.033*** |  |  |  |  |  |  |

A&E, Accident and Emergency; RAMP-DM, Risk Assessment and Management Programme – Diabetes Mellitus; SD, Standard deviation.

Notes:

* Significant at 0.05 level by generalized linear model with Poisson family, log link and robust standard errors.

Patients with full 12-month follow-up in each year were included.

**Table S11. Sensitivity analysis of ten-year direct medical costs under varying discounted rates and RAMP-DM recurrent costs**

a) Costs discounted at 3% annually

| US$ | RAMP-DM participants | | | Usual care patients | Difference | |
| --- | --- | --- | --- | --- | --- | --- |
|  | Health service utilization (95% CI) | RAMP-DM programme cost  (95% CI) | Total (95% CI) | Total (Health service utilization) (95% CI) | Health service utilization (95% CI) | Total (95% CI) |
| 1^st^ year | 1,390 (1,319,1,461) | 74 (73.1,74.6) | 1,464 (1,393,1,534) | 2,979 (2,845,3,112) | -1,589 (-1,738,-1,439) | -1,515 (-1,665,-1,365) |
| 2^nd^ year | 1,620 (1,531,1,708) | 32 (31.7,32.9) | 1,652 (1,563,1,741) | 3,218 (3,064,3,372) | -1,598 (-1,772,-1,424) | -1,566 (-1,740,-1,392) |
| 3^rd^ year | 1,644 (1,555,1,733) | 30 (29.3,30.5) | 1,674 (1,585,1,763) | 2,999 (2,846,3,152) | -1,355 (-1,528,-1,183) | -1,325 (-1,498,-1,153) |
| 4^th^ year | 1,778 (1,676,1,879) | 28 (27.4,28.4) | 1,806 (1,704,1,907) | 2,749 (2,603,2,895) | -971 (-1,145,-797) | -943 (-1,117,-769) |
| 5^th^ year | 1,865 (1,754,1,976) | 23 (23.0,24.0) | 1,889 (1,778,1,999) | 2,647 (2,509,2,785) | -782 (-957,-607) | -758 (-933,-583) |
| 6^th^ year | 1,943 (1,841,2,046) | 24 (23.1,24.1) | 1,967 (1,864,2,069) | 2,723 (2,572,2,874) | -780 (-957,-603) | -756 (-933,-579) |
| 7^th^ year | 2,035 (1,925,2,146) | 21 (20.8,21.9) | 2,057 (1,947,2,167) | 2,746 (2,594,2,898) | -711 (-894,-527) | -689 (-872,-506) |
| 8^th^ year | 2,166 (2,056,2,276) | 20 (19.8,20.8) | 2,186 (2,076,2,297) | 2,794 (2,643,2,945) | -628 (-811,-445) | -608 (-790,-425) |
| 9^th^ year | 2,191 (2,083,2,298) | 19 (18.4,19.3) | 2,209 (2,102,2,317) | 2,893 (2,730,3,055) | -702 (-890,-514) | -683 (-871,-495) |
| 10^th^ year | 2,653 (2,333,2,973) | 17 (16.0,18.0) | 2,670 (2,350,2,990) | 3,057 (2,870,3,245) | -404 (-782,-27) | -387 (-765,-10) |
| **Total^†^** | **19,284 (18,744,19,847)** | **289 (285,292)** | **19,573 (19,032,20, 136)** | **28,804 (28,082,29,572)** | **-9,520 (-10,462,-8,608)** | **-9,231 (-10,173,-8,320)** |

b) Costs discounted at 5% annually

| US$ | RAMP-DM participants | | | Usual care patients | Difference | |
| --- | --- | --- | --- | --- | --- | --- |
|  | Health service utilization (95% CI) | RAMP-DM programme cost (95% CI) | Total (95% CI) | Total (Health service utilization) (95% CI) | Health service utilization (95% CI) | Total (95% CI) |
| 1^st^ year | 1,363 (1,294,1,433) | 72 (71.7,73.2) | 1,436 (1,366,1,505) | 2,922 (2,791,3,053) | -1,559 (-1,705,-1,412) | -1,486 (-1,633,-1,339) |
| 2^nd^ year | 1,559 (1,473,1,644) | 31 (30.5,31.7) | 1,590 (1,504,1,675) | 3,096 (2,948,3,245) | -1,538 (-1,705,-1,370) | -1,507 (-1,674,-1,339) |
| 3^rd^ year | 1,552 (1,468,1,636) | 28 (27.6,28.8) | 1,580 (1,496,1,664) | 2,831 (2,686,2,976) | -1,279 (-1,442,-1,117) | -1,251 (-1,414,-1,089) |
| 4^th^ year | 1,646 (1,552,1,740) | 26 (25.3,26.3) | 1,672 (1,578,1,766) | 2,545 (2,410,2,681) | -899 (-1,061,-738) | -873 (-1,035,-712) |
| 5^th^ year | 1,694 (1,593,1,795) | 21 (20.9,21.8) | 1,715 (1,615,1,816) | 2,404 (2,279,2,530) | -710 (-869,-551) | -689 (-848,-530) |
| 6^th^ year | 1,731 (1,640,1,823) | 21 (20.6,21.5) | 1,753 (1,661,1,844) | 2,426 (2,292,2,561) | -695 (-853,-537) | -674 (-832,-516) |
| 7^th^ year | 1,779 (1,683,1,875) | 19 (18.2,19.1) | 1,798 (1,702,1,894) | 2,400 (2,268,2,533) | -621 (-781,-461) | -602 (-762,-442) |
| 8^th^ year | 1,857 (1,762,1,952) | 17 (17.0,17.9) | 1,875 (1,780,1,969) | 2,396 (2,266,2,525) | -538 (-695,-382) | -521 (-678,-364) |
| 9^th^ year | 1,842 (1,752,1,933) | 16 (15.5,16.3) | 1,858 (1,768,1,949) | 2,433 (2,297,2,569) | -590 (-748,-433) | -575 (-732,-417) |
| 10^th^ year | 2,189 (1,925,2,453) | 14 (13.2,14.8) | 2,203 (1,939,2,467) | 2,523 (2,368,2,677) | -334 (-645,-22) | -320 (-631,-8) |
| **Total^†^** | **17,213 (16,739,17,702)** | **266 (263,269)** | **17,479 (17,006,17,969)** | **25,976 (25,322,26,665)** | **-8,764 (-9.598,-7,948)** | **-8,498 (-9,331,-7,682)** |

c) Varying RAMP-DM recurrent costs by -20%

| US$ | RAMP-DM participants | | | Usual care patients | Difference | |  |
| --- | --- | --- | --- | --- | --- | --- | --- |
|  | Health service utilization (95% CI) | RAMP-DM programme cost (95% CI) | Total (95% CI) | Total (Health service utilization) (95% CI) | Health service utilization (95% CI) | Total (95% CI) | |
| 1^st^ year | 1,431 (1,359,1,504) | 64 (63.0,64.3) | 1,495 (1,422,1,568) | 3,068 (2,930,3,206) | -1,636 (-1,791,-1,482) | -1,573 (-1,727,-1,419) | |
| 2^nd^ year | 1,718 (1,624,1,812) | 27 (26.9,28.0) | 1,746 (1,652,1,840) | 3,414 (3,250,3,577) | -1,695 (-1,880,-1,511) | -1,668 (-1,853,-1,483) | |
| 3^rd^ year | 1,796 (1,699,1,893) | 26 (25.6,26.7) | 1,822 (1,725,1,919) | 3,277 (3,110,3,445) | -1,481 (-1,669,-1,293) | -1,455 (-1,643,-1,267) | |
| 4^th^ year | 2,001 (1,887,2,115) | 25 (24.6,25.6) | 2,026 (1,912,2,140) | 3,094 (2,930,3,259) | -1,093 (-1,289,-897) | -1,068 (-1,264,-872) | |
| 5^th^ year | 2,162 (2,034,2,291) | 22 (21.3,22.3) | 2,184 (2,055,2,312) | 3,068 (2,908,3,229) | -906 (-1,109,-703) | -884 (-1,087,-681) | |
| 6^th^ year | 2,320 (2,198,2,443) | 23 (22.0,23.1) | 2,343 (2,221,2,465) | 3,251 (3,072,3,431) | -931 (-1,143,-720) | -909 (-1,120,-697) | |
| 7^th^ year | 2,503 (2,368,2,639) | 21 (20.5,21.5) | 2,524 (2,389,2,660) | 3,377 (3,191,3,564) | -874 (-1,099,-649) | -853 (-1,078,-628) | |
| 8^th^ year | 2,744 (2,604,2,884) | 21 (20.1,21.1) | 2,764 (2,625,2,904) | 3,539 (3,349,3,730) | -796 (-1,027,-564) | -775 (-1,006,-544) | |
| 9^th^ year | 2,858 (2,718,2,998) | 20 (19.2,20.2) | 2,878 (2,738,3,018) | 3,774 (3,563,3,986) | -916 (-1,161,-671) | -896 (-1,141,-651) | |
| 10^th^ year | 3,565 (3,135,3,996) | 18 (17.2,19.3) | 3,584 (3,153,4,014) | 4,109 (3,857,4,361) | -543 (-1,051,-36) | -525 (-1,032,-18) | |
| **Total^†^** | **23,100 (22,433,23,810)** | **266 (263,269)** | **23,366 (22,699,24,075)** | **33,973 (33,107,34,882)** | **-10,872 (-12,021,-9,762)** | **-10,606 (-11,754,-9,497)** | |

d) Varying RAMP-DM recurrent costs by -10%

| US$ | RAMP-DM participants | | | Usual care patients | Difference | |  |
| --- | --- | --- | --- | --- | --- | --- | --- |
|  | Health service utilization (95% CI) | RAMP-DM programme cost (95% CI) | Total (95% CI) | Total (Health service utilization) (95% CI) | Health service utilization (95% CI) | Total (95% CI) | |
| 1^st^ year | 1,431 (1,359,1,504) | 70 (69.1,70.5) | 1,501 (1,429,1,574) | 3,068 (2,930,3,206) | -1,636 (-1,791,-1,482) | -1,567 (-1,721,-1,412) | |
| 2^nd^ year | 1,718 (1,624,1,812) | 31 (30.3,31.4) | 1,749 (1,655,1,843) | 3,414 (3,250,3,577) | -1,695 (-1,880,-1,511) | -1,665 (-1,849,-1,480) | |
| 3^rd^ year | 1,796 (1,699,1,893) | 29 (28.8,30.0) | 1,826 (1,728,1,923) | 3,277 (3,110,3,445) | -1,481 (-1,669,-1,293) | -1,452 (-1,640,-1,263) | |
| 4^th^ year | 2,001 (1,887,2,115) | 28 (27.7,28.8) | 2,029 (1,915,2,144) | 3,094 (2,930,3,259) | -1,093 (-1,289,-897) | -1,065 (-1,261,-869) | |
| 5^th^ year | 2,162 (2,034,2,291) | 25 (24.0,25.0) | 2,187 (2,058,2,315) | 3,068 (2,908,3,229) | -906 (-1,109,-703) | -882 (-1,085,-679) | |
| 6^th^ year | 2,320 (2,198,2,443) | 25 (24.8,25.9) | 2,346 (2,223,2,468) | 3,251 (3,072,3,431) | -931 (-1,143,-720) | -906 (-1,117,-694) | |
| 7^th^ year | 2,503 (2,368,2,639) | 24 (23.0,24.2) | 2,527 (2,392,2,662) | 3,377 (3,191,3,564) | -874 (-1,099,-649) | -850 (-1,076,-625) | |
| 8^th^ year | 2,744 (2,604,2,884) | 23 (22.6,23.7) | 2,767 (2,627,2,907) | 3,539 (3,349,3,730) | -796 (-1,027,-564) | -772 (-1,004,-541) | |
| 9^th^ year | 2,858 (2,718,2,998) | 22 (21.6,22.7) | 2,880 (2,740,3,020) | 3,774 (3,563,3,986) | -916 (-1,161,-671) | -894 (-1,139,-649) | |
| 10^th^ year | 3,565 (3,135,3,996) | 21 (19.4,21.7) | 3,586 (3,155,4,017) | 4,109 (3,857,4,361) | -543 (-1,051,-36) | -523 (-1,030,-16) | |
| **Total^†^** | **23,100 (22,433,23,810)** | **298 (294,301)** | **23,398 (22,730,24,107)** | **33,973 (33,107,34,882)** | **-10,872 (-12,021,-9,762)** | **-10,575 (-11,723,-9,466)** | |

e) Varying RAMP-DM recurrent costs by +10%

| US$ | RAMP-DM participants | | | Usual care patients | Difference | |  |
| --- | --- | --- | --- | --- | --- | --- | --- |
|  | Health service utilization (95% CI) | RAMP-DM programme cost (95% CI) | Total (95% CI) | Total (Health service utilization) (95% CI) | Health service utilization (95% CI) | Total (95% CI) | |
| 1^st^ year | 1,431 (1,359,1,504) | 82 (81.4,83.1) | 1,514 (1,441,1,587) | 3,068 (2,930,3,206) | -1,636 (-1,791,-1,482) | -1,554 (-1,708,-1,400) | |
| 2^nd^ year | 1,718 (1,624,1,812) | 38 (37.0,38.4) | 1,756 (1,662,1,850) | 3,414 (3,250,3,577) | -1,695 (-1,880,-1,511) | -1,658 (-1,842,-1,473) | |
| 3^rd^ year | 1,796 (1,699,1,893) | 36 (35.2,36.7) | 1,832 (1,735,1,929) | 3,277 (3,110,3,445) | -1,481 (-1,669,-1,293) | -1,445 (-1,633,-1,257) | |
| 4^th^ year | 2,001 (1,887,2,115) | 35 (33.9,35.2) | 2,036 (1,921,2,150) | 3,094 (2,930,3,259) | -1,093 (-1,289,-897) | -1,058 (-1,255,-862) | |
| 5^th^ year | 2,162 (2,034,2,291) | 30 (29.3,30.6) | 2,192 (2,064,2,321) | 3,068 (2,908,3,229) | -906 (-1,109,-703) | -876 (-1,079,-673) | |
| 6^th^ year | 2,320 (2,198,2,443) | 31 (30.3,31.7) | 2,351 (2,229,2,474) | 3,251 (3,072,3,431) | -931 (-1,143,-720) | -900 (-1,112,-689) | |
| 7^th^ year | 2,503 (2,368,2,639) | 29 (28.2,29.6) | 2,532 (2,397,2,668) | 3,377 (3,191,3,564) | -874 (-1,099,-649) | -845 (-1,070,-620) | |
| 8^th^ year | 2,744 (2,604,2,884) | 28 (27.6,29.0) | 2,772 (2,632,2,912) | 3,539 (3,349,3,730) | -796 (-1,027,-564) | -767 (-999,-536) | |
| 9^th^ year | 2,858 (2,718,2,998) | 27 (26.4,27.8) | 2,885 (2,745,3,025) | 3,774 (3,563,3,986) | -916 (-1,161,-671) | -889 (-1,134,-644) | |
| 10^th^ year | 3,565 (3,135,3,996) | 25 (23.7,26.6) | 3,591 (3,160,4,021) | 4,109 (3,857,4,361) | -543 (-1,051,-36) | -518 (-1,025,-11) | |
| **Total^†^** | **23,100 (22,433,23,810)** | **361 (357,365)** | **23,461 (22,794,24,170)** | **33,973 (33,107,34,882)** | **-10,872 (-12,021,-9,762)** | **-10,512 (-11,660,-9,403)** | |

f) Varying RAMP-DM recurrent costs by +20%

| US$ | RAMP-DM participants | | | Usual care patients | Difference | |  |
| --- | --- | --- | --- | --- | --- | --- | --- |
|  | Health service utilization (95% CI) | RAMP-DM programme cost (95% CI) | Total (95% CI) | Total (Health service utilization) (95% CI) | Health service utilization (95% CI) | Total (95% CI) | |
| 1^st^ year | 1,431 (1,359,1,504) | 88 (87.6,89.4) | 1,520 (1,447,1,593) | 3,068 (2,930,3,206) | -1,636 (-1,791,-1,482) | -1,548 (-1,702,-1,394) | |
| 2^nd^ year | 1,718 (1,624,1,812) | 41 (40.4,41.9) | 1,759 (1,665,1,854) | 3,414 (3,250,3,577) | -1,695 (-1,880,-1,511) | -1,654 (-1,839,-1,470) | |
| 3^rd^ year | 1,796 (1,699,1,893) | 39 (38.4,40.0) | 1,835 (1,738,1,933) | 3,277 (3,110,3,445) | -1,481 (-1,669,-1,293) | -1,442 (-1,630,-1,254) | |
| 4^th^ year | 2,001 (1,887,2,115) | 38 (37.0,38.4) | 2,039 (1,924,2,153) | 3,094 (2,930,3,259) | -1,093 (-1,289,-897) | -1,055 (-1,251,-859) | |
| 5^th^ year | 2,162 (2,034,2,291) | 33 (32.0,33.4) | 2,195 (2,066,2,323) | 3,068 (2,908,3,229) | -906 (-1,109,-703) | -874 (-1,077,-671) | |
| 6^th^ year | 2,320 (2,198,2,443) | 34 (33.1,34.6) | 2,354 (2,232,2,476) | 3,251 (3,072,3,431) | -931 (-1,143,-720) | -897 (-1,109,-686) | |
| 7^th^ year | 2,503 (2,368,2,639) | 32 (30.7,32.3) | 2,535 (2,400,2,670) | 3,377 (3,191,3,564) | -874 (-1,099,-649) | -842 (-1,068,-617) | |
| 8^th^ year | 2,744 (2,604,2,884) | 31 (30.1,31.6) | 2,775 (2,635,2,915) | 3,539 (3,349,3,730) | -796 (-1,027,-564) | -765 (-996,-533) | |
| 9^th^ year | 2,858 (2,718,2,998) | 30 (28.8,30.3) | 2,888 (2,748,3,028) | 3,774 (3,563,3,986) | -916 (-1,161,-671) | -886 (-1,131,-642) | |
| 10^th^ year | 3,565 (3,135,3,996) | 27 (25.8,29.0) | 3,593 (3,162,4,023) | 4,109 (3,857,4,361) | -543 (-1,051,-36) | -516 (-1,023,-9) | |
| **Total^†^** | **23,100 (22,433,23,810)** | **392 (388,397)** | **23,493 (22,825,24,202)** | **33,973 (33,107,34,882)** | **-10,872 (-12,021,-9,762)** | **-10,480 (-11,628,-9,372)** | |

CI, Confidence interval.

Note:

† The 95% CI for the total ten-year costs were constructed using bootstrapping with a simulation of 10,000 patients.

**Table S12. Sensitivity analysis of ten-year cost-effectiveness of RAMP-DM under varying discounted rates and recurrent costs**

a) Costs and clinical outcomes discounted at 3% annually

|  | Incremental cost | | Incremental effectiveness | Incremental cost-effectiveness ratio | |
| --- | --- | --- | --- | --- | --- |
|  | (1) | (2) |  | (1) | (2) |
|  | RAMP-DM cost, US$ (95% CI^†^) | Total public direct medical cost, US$ (95% CI^†^) | Event prevented per 100 patients (95% CI) | Programme cost per event prevented, US$ (95% CI^†^) | Total public direct medical cost per event prevented, US$ (95% CI) |
| Any complication | 289 (285,292) | -9,231 (-10,173,-8,320) | 11.4 (10.5,12.2) | 2,535 (2,358,2,740) | Cost-saving |
| CVD | 289 (285,292) | -9,231 (-10,173,-8,320) | 11.2 (10.4,12.0) | 2,578 (2,407,2,774) | Cost-saving |
| CHD | 289 (285,292) | -9,231 (-10,173,-8,320) | 6.3 (5.7,6.9) | 4,565 (4,164,5,034) | Cost-saving |
| Heart failure | 289 (285,292) | -9,231 (-10,173,-8,320) | 4.0 (3.5,4.4) | 7,271 (6,505,8,238) | Cost-saving |
| Stroke | 289 (285,292) | -9,231 (-10,173,-8,320) | 4.8 (4.2,5.4) | 6,050 (5,377,6,901) | Cost-saving |
| PVD | 289 (285,292) | -9,231 (-10,173,-8,320) | 0.8 (0.6,1.1) | 33,961 (26,840,46,458) | Cost-saving |
| Any microvascular complication | 289 (285,292) | -9,231 (-10,173,-8,320) | 3.0 (2.4,3.5) | 9,750 (8,150,12,055) | Cost-saving |
| NPDR | 289 (285,292) | -9,231 (-10,173,-8,320) | 0.004 (-0.4,0.4) | 6,960,634 (Not reported^‡^) | Cost-saving |
| STDR | 289 (285,292) | -9,231 (-10,173,-8,320) | 0.9 (0.6,1.1) | 32,889 (25,711,44,862) | Cost-saving |
| Diabetic neuropathy | 289 (285,292) | -9,231 (-10,173,-8,320) | 0.6 (0.4,0.8) | 47,816 (35,419,73,675) | Cost-saving |
| ESRD | 289 (285,292) | -9,231 (-10,173,-8,320) | 2.7 (2.3,3.1) | 10,555 (9,196,12,359) | Cost-saving |
| All-cause mortality | 289 (285,292) | -9,231 (-10,173,-8,320) | 14.4 (13.7,15.2) | 2,001 (1,904,2,109) | Cost-saving |

b) Costs and clinical outcomes discounted at 5% annually

|  | Incremental cost | | Incremental effectiveness | Incremental cost-effectiveness ratio | |
| --- | --- | --- | --- | --- | --- |
|  | (1) | (2) |  | (1) | (2) |
|  | RAMP-DM cost, US$ (95% CI^†^) | Total public direct medical cost, US$ (95% CI^†^) | Event prevented per 100 patients (95% CI) | Programme cost per event prevented, US$ (95% CI^†^) | Total public direct medical cost per event prevented, US$ (95% CI) |
| Any complication | 266 (263,269) | -8,498 (-9,331,-7,682) | 11.0 (10.2,11.8) | 2,422 (2,258,2,610) | Cost-saving |
| CVD | 266 (263,269) | -8,498 (-9,331,-7,682) | 10.8 (10.0,11.5) | 2,471 (2,312,2,651) | Cost-saving |
| CHD | 266 (263,269) | -8,498 (-9,331,-7,682) | 6.0 (5.5,6.6) | 4,413 (4,036,4,850) | Cost-saving |
| Heart failure | 266 (263,269) | -8,498 (-9,331,-7,682) | 3.7 (3.3,4.2) | 7,144 (6,409,8,075) | Cost-saving |
| Stroke | 266 (263,269) | -8,498 (-9,331,-7,682) | 4.6 (4.0,5.1) | 5,840 (5,214,6,625) | Cost-saving |
| PVD | 266 (263,269) | -8,498 (-9,331,-7,682) | 0.8 (0.6,1.0) | 33,205 (26,365,44,909) | Cost-saving |
| Any microvascular complication | 266 (263,269) | -8,498 (-9,331,-7,682) | 2.8 (2.2,3.3) | 9,619 (8,056,11,860) | Cost-saving |
| NPDR | 266 (263,269) | -8,498 (-9,331,-7,682) | 0.03 (-0.3,0.4) | 840,981 (-2,357,409,2,147,122) | Cost-saving |
| STDR | 266 (263,269) | -8,498 (-9,331,-7,682) | 0.8 (0.6,1.0) | 32,539 (25,504,44,161) | Cost-saving |
| Diabetic neuropathy | 266 (263,269) | -8,498 (-9,331,-7,682) | 0.6 (0.4,0.8) | 47,154 (35,042,71,981) | Cost-saving |
| ESRD | 266 (263,269) | -8,498 (-9,331,-7,682) | 2.5 (2.1,2.9) | 10,644 (9,290,12,424) | Cost-saving |
| All-cause mortality | 266 (263,269) | -8,498 (-9,331,-7,682) | 13.4 (12.7,14.1) | 1,982 (1,887,2,086) | Cost-saving |

c) Varying RAMP-DM recurrent costs by -20%

|  | Incremental cost | | Incremental effectiveness | Incremental cost-effectiveness ratio | |
| --- | --- | --- | --- | --- | --- |
|  | (1) | (2) |  | (1) | (2) |
|  | RAMP-DM cost, US$ (95% CI^†^) | Total public direct medical cost, US$ (95% CI^†^) | Event prevented per 100 patients (95% CI) | Programme cost per event prevented, US$ (95% CI^†^) | Total public direct medical cost per event prevented, US$ (95% CI) |
| Any complication | 266 (263,269) | -10,606 (-11,754,-9,497) | 12.1 (11.1,13.0) | 2,209 (2,046,2,399) | Cost-saving |
| CVD | 266 (263,269) | -10,606 (-11,754,-9,497) | 11.9 (11.0,12.8) | 2,234 (2,076,2,418) | Cost-saving |
| CHD | 266 (263,269) | -10,606 (-11,754,-9,497) | 6.8 (6.2,7.5) | 3,901 (3,541,4,323) | Cost-saving |
| Heart failure | 266 (263,269) | -10,606 (-11,754,-9,497) | 4.4 (3.9,4.9) | 6,046 (5,383,6,881) | Cost-saving |
| Stroke | 266 (263,269) | -10,606 (-11,754,-9,497) | 5.1 (4.5,5.8) | 5,181 (4,571,5,976) | Cost-saving |
| PVD | 266 (263,269) | -10,606 (-11,754,-9,497) | 0.9 (0.7,1.2) | 28,437 (22,240,39,680) | Cost-saving |
| Any microvascular complication | 266 (263,269) | -10,606 (-11,754,-9,497) | 3.3 (2.6,4.0) | 8,045 (6,698,10,008) | Cost-saving |
| NPDR | 266 (263,269) | -10,606 (-11,754,-9,497) | -0.05 (-0.5,0.4) | Not effective | Not effective |
| STDR | 266 (263,269) | -10,606 (-11,754,-9,497) | 1.0 (0.7,1.3) | 27,023 (21,024,37,178) | Cost-saving |
| Diabetic neuropathy | 266 (263,269) | -10,606 (-11,754,-9,497) | 0.7 (0.4,0.9) | 39,445 (29,004,61,449) | Cost-saving |
| ESRD | 266 (263,269) | -10,606 (-11,754,-9,497) | 3.2 (2.7,3.6) | 8,419 (7,303,9,915) | Cost-saving |
| All-cause mortality | 266 (263,269) | -10,606 (-11,754,-9,497) | 16.2 (15.3,17.1) | 1,644 (1,561,1,737) | Cost-saving |

d) Varying RAMP-DM recurrent costs by -10%

|  | Incremental cost | | Incremental effectiveness | Incremental cost-effectiveness ratio | |
| --- | --- | --- | --- | --- | --- |
|  | (1) | (2) |  | (1) | (2) |
|  | RAMP-DM cost, US$ (95% CI^†^) | Total public direct medical cost, US$ (95% CI^†^) | Event prevented per 100 patients (95% CI) | Programme cost per event prevented, US$ (95% CI^†^) | Total public direct medical cost per event prevented, US$ (95% CI) |
| Any complication | 298 (294,301) | -10,575 (-11,723,-9,466) | 12.1 (11.1,13.0) | 2,471 (2,288,2,683) | Cost-saving |
| CVD | 298 (294,301) | -10,575 (-11,723,-9,466) | 11.9 (11.0,12.8) | 2,499 (2,322,2,705) | Cost-saving |
| CHD | 298 (294,301) | -10,575 (-11,723,-9,466) | 6.8 (6.2,7.5) | 4,363 (3,961,4,835) | Cost-saving |
| Heart failure | 298 (294,301) | -10,575 (-11,723,-9,466) | 4.4 (3.9,4.9) | 6,762 (6,020,7,696) | Cost-saving |
| Stroke | 298 (294,301) | -10,575 (-11,723,-9,466) | 5.1 (4.5,5.8) | 5,795 (5,112,6,684) | Cost-saving |
| PVD | 298 (294,301) | -10,575 (-11,723,-9,466) | 0.9 (0.7,1.2) | 31,806 (24,873,44,380) | Cost-saving |
| Any microvascular complication | 298 (294,301) | -10,575 (-11,723,-9,466) | 3.3 (2.6,4.0) | 8,998 (7,491,11,194) | Cost-saving |
| NPDR | 298 (294,301) | -10,575 (-11,723,-9,466) | -0.05 (-0.5,0.4) | Not effective | Not effective |
| STDR | 298 (294,301) | -10,575 (-11,723,-9,466) | 1.0 (0.7,1.3) | 30,225 (23,514,41,584) | Cost-saving |
| Diabetic neuropathy | 298 (294,301) | -10,575 (-11,723,-9,466) | 0.7 (0.4,0.9) | 44,118 (32,441,68,733) | Cost-saving |
| ESRD | 298 (294,301) | -10,575 (-11,723,-9,466) | 3.2 (2.7,3.6) | 9,416 (8,168,11,090) | Cost-saving |
| All-cause mortality | 298 (294,301) | -10,575 (-11,723,-9,466) | 16.2 (15.3,17.1) | 1,838 (1,746,1,942) | Cost-saving |

e) Varying RAMP-DM recurrent costs by +10%

|  | Incremental cost | | Incremental effectiveness | Incremental cost-effectiveness ratio | |
| --- | --- | --- | --- | --- | --- |
|  | (1) | (2) |  | (1) | (2) |
|  | RAMP-DM cost, US$ (95% CI^†^) | Total public direct medical cost, US$ (95% CI^†^) | Event prevented per 100 patients (95% CI) | Programme cost per event prevented, US$ (95% CI^†^) | Total public direct medical cost per event prevented, US$ (95% CI) |
| Any complication | 361 (357,365) | -10,512 (-11,660,-9,403) | 12.1 (11.1,13.0) | 2,994 (2,773,3,251) | Cost-saving |
| CVD | 361 (357,365) | -10,512 (-11,660,-9,403) | 11.9 (11.0,12.8) | 3,029 (2,814,3,278) | Cost-saving |
| CHD | 361 (357,365) | -10,512 (-11,660,-9,403) | 6.8 (6.2,7.5) | 5,287 (4,800,5,860) | Cost-saving |
| Heart failure | 361 (357,365) | -10,512 (-11,660,-9,403) | 4.4 (3.9,4.9) | 8,195 (7,295,9,327) | Cost-saving |
| Stroke | 361 (357,365) | -10,512 (-11,660,-9,403) | 5.1 (4.5,5.8) | 7,023 (6,195,8,101) | Cost-saving |
| PVD | 361 (357,365) | -10,512 (-11,660,-9,403) | 0.9 (0.7,1.2) | 38,544 (30,139,53,781) | Cost-saving |
| Any microvascular complication | 361 (357,365) | -10,512 (-11,660,-9,403) | 3.3 (2.6,4.0) | 10,904 (9,078,13,564) | Cost-saving |
| NPDR | 361 (357,365) | -10,512 (-11,660,-9,403) | -0.05 (-0.5,0.4) | Not effective | Not effective |
| STDR | 361 (357,365) | -10,512 (-11,660,-9,403) | 1.0 (0.7,1.3) | 36,627 (28,495,50,395) | Cost-saving |
| Diabetic neuropathy | 361 (357,365) | -10,512 (-11,660,-9,403) | 0.7 (0.4,0.9) | 53,464 (39,314,83,301) | Cost-saving |
| ESRD | 361 (357,365) | -10,512 (-11,660,-9,403) | 3.2 (2.7,3.6) | 11,411 (9,898,13,440) | Cost-saving |
| All-cause mortality | 361 (357,365) | -10,512 (-11,660,-9,403) | 16.2 (15.3,17.1) | 2,228 (2,116,2,354) | Cost-saving |

f) Varying RAMP-DM recurrent costs by +20%

|  | Incremental cost | | Incremental effectiveness | Incremental cost-effectiveness ratio | |
| --- | --- | --- | --- | --- | --- |
|  | (1) | (2) |  | (1) | (2) |
|  | RAMP-DM cost, US$ (95% CI^†^) | Total public direct medical cost, US$ (95% CI^†^) | Event prevented per 100 patients (95% CI) | Programme cost per event prevented, US$ (95% CI^†^) | Total public direct medical cost per event prevented, US$ (95% CI) |
| Any complication | 392 (388,397) | -10,480 (-11,628,-9,372) | 12.1 (11.1,13.0) | 3,256 (3,015,3,536) | Cost-saving |
| CVD | 392 (388,397) | -10,480 (-11,628,-9,372) | 11.9 (11.0,12.8) | 3,293 (3,060,3,564) | Cost-saving |
| CHD | 392 (388,397) | -10,480 (-11,628,-9,372) | 6.8 (6.2,7.5) | 5,749 (5,219,6,372) | Cost-saving |
| Heart failure | 392 (388,397) | -10,480 (-11,628,-9,372) | 4.4 (3.9,4.9) | 8,911 (7,933,10,143) | Cost-saving |
| Stroke | 392 (388,397) | -10,480 (-11,628,-9,372) | 5.1 (4.5,5.8) | 7,637 (6,736,8,809) | Cost-saving |
| PVD | 392 (388,397) | -10,480 (-11,628,-9,372) | 0.9 (0.7,1.2) | 41,912 (32,773,58,481) | Cost-saving |
| Any microvascular complication | 392 (388,397) | -10,480 (-11,628,-9,372) | 3.3 (2.6,4.0) | 11,857 (9,871,14,749) | Cost-saving |
| NPDR | 392 (388,397) | -10,480 (-11,628,-9,372) | -0.05 (-0.5,0.4) | Not effective | Not effective |
| STDR | 392 (388,397) | -10,480 (-11,628,-9,372) | 1.0 (0.7,1.3) | 39,828 (30,986,54,801) | Cost-saving |
| Diabetic neuropathy | 392 (388,397) | -10,480 (-11,628,-9,372) | 0.7 (0.4,0.9) | 58,137 (42,751,90,586) | Cost-saving |
| ESRD | 392 (388,397) | -10,480 (-11,628,-9,372) | 3.2 (2.7,3.6) | 12,408 (10,763,14,614) | Cost-saving |
| All-cause mortality | 392 (388,397) | -10,480 (-11,628,-9,372) | 16.2 (15.3,17.1) | 2,422 (2,301,2,560) | Cost-saving |

CHD, coronary heart disease; CI, confidence interval; CVD, cardiovascular disease; ESRD, end-stage renal disease; PVD, peripheral vascular disease; RAMP-DM, Risk Assessment and Management Programme – Diabetes Mellitus; STDR, sight-threatening diabetic retinopathy.

Notes: † The 95% CI was constructed using bootstrapping with a simulation of 10,000 patients; ‡ The 95% CI was not reported due to the small value of number of events prevented.

**Figure S1. Tornado diagram of one-way sensitivity analyses on factors affecting direct medical costs**

RAMP-DM, Risk Assessment and Management Programme – Diabetes Mellitus.

Notes:

Only top 30 most influential factors are presented.

Healthcare service utilization and RAMP-DM costs were varied within the 95% confidence intervals and across the range observed in all seven clusters of the Hospital Authority, respectively.
